# Structural and spectroscopic characterization of RufO indicates a new biological role in rufomycin biosynthesis

**DOI:** 10.1016/j.jbc.2023.105049

**Published:** 2023-07-13

**Authors:** Stephanie Jordan, Bingnan Li, Ephrahime Traore, Yifei Wu, Remigio Usai, Aimin Liu, Zhong-Ru Xie, Yifan Wang

**Affiliations:** 1 Department of Chemistry, University of Georgia, Athens, Georgia, USA; 2 Department of Chemistry, University of Texas at San Antonio, San Antonio, Texas, USA; 3 School of Electrical and Computer Engineering, University of Georgia, Athens, Georgia, USA

**Keywords:** heme-dependent catalysis, tyrosyl nitration, cytochrome P450, nonribosomal peptide synthesis, rufomycin

## Abstract

Rufomycins constitute a class of cyclic heptapeptides isolated from actinomycetes. They are secondary metabolites that show promising treatment against *Mycobacterium tuberculosis* infections by inhibiting a novel drug target. Several nonproteinogenic amino acids are integrated into rufomycins, including a conserved 3-nitro-tyrosine. RufO, a cytochrome P450 (CYP)-like enzyme, was proposed to catalyze the formation of 3-nitro-tyrosine in the presence of O_2_ and NO. To define its biological function, the interaction between RufO and the proposed substrate tyrosine is investigated using various spectroscopic methods that are sensitive to the structural change of a heme center. However, a low- to high-spin state transition and a dramatic increase in the redox potential that are commonly found in CYPs upon ligand binding have not been observed. Furthermore, a 1.89-Å crystal structure of RufO shows that the enzyme has flexible surface regions, a wide-open substrate access tunnel, and the heme center is largely exposed to solvent. Comparison with a closely related nitrating CYP reveals a spacious and hydrophobic distal pocket in RufO, which is incapable of stabilizing a free amino acid. Molecular docking validates the experimental data and proposes a possible substrate. Collectively, our results disfavor tyrosine as the substrate of RufO and point to the possibility that the nitration occurs during or after the assembly of the peptides. This study indicates a new function of the unique nitrating enzyme and provides insights into the biosynthesis of nonribosomal peptides.

Rufomycins, also known as ilamycins, are cyclopeptides first identified in 1962 from *Streptomyces* (1, 2). Since their discovery, rufomycins have drawn much attention for their extraordinary antitubercular activities (2, 3, 4). Multiple naturally occurring rufomycins have been isolated thus far, and the list is continuously expanding (5, 6). Some rufomycins exhibit excellent inhibitory effects on one of the world’s deadliest infectious agents, *Mycobacterium tuberculosis*, outcompeting first-line tuberculosis drugs such as isoniazid and rifampin (7, 8). Recently, the inhibitory mechanism of rufomycins has become more clear. Molecular studies elucidated that rufomycins bind to an unfoldase ClpC1 at the *N*-terminal domain (8, 9), which triggers the formation of higher-order ClpC1 oligomers or aggregates and thus hampers ClpC1’s coupling with a caseinolytic protease complex (10). As a result, the unsuccessfully assembled protease machinery fails to maintain protein homeostasis and severely damages the viability of *Mycobacterium tuberculosis*. ClpC1 is an emerging drug target, which makes rufomycins promising compounds for tuberculosis treatment, especially for cases caused by drug-resistant strains.

The general structure of rufomycins has been known for decades (Fig. 1*A*), but the biosynthetic pathway was only recently characterized (11, 12, 13). A nonproteinogenic amino acid, 3-nitro-tyrosine (3-NO_2_-Tyr), is prominent and strictly conserved in rufomycins, although the role of the nitro group in the ClpC1-targeted inhibition remains elusive. It was proposed by Tomita et al. that the 3-NO_2_-Tyr is derived from the direct nitration of Tyr catalyzed by RufO in the presence of oxygen and nitric oxide (NO) (Fig. 1*B*), and a dedicated nitric oxide synthase, encoded by *rufN* in the same putative operon as *rufO*, supplies the required NO (11). Once Tyr is nitrated, a heptamodular nonribosomal peptide synthase (NRPS) RufT recruits 3-NO_2_-Tyr through the third module to assemble the cyclized heptapeptide. The same study also reported that deleting the *rufO* gene disrupted the rufomycin biosynthesis, but an *in vitro* enzymatic assay using recombinant RufO only showed marginal activity. The same biological function was also proposed for IlaN, a RufO homolog, by an independent study (13). Unfortunately, there is no follow-up study on the system after the first identification of RufO and the molecular details of how RufO functions are unclear.

**Figure 1:**
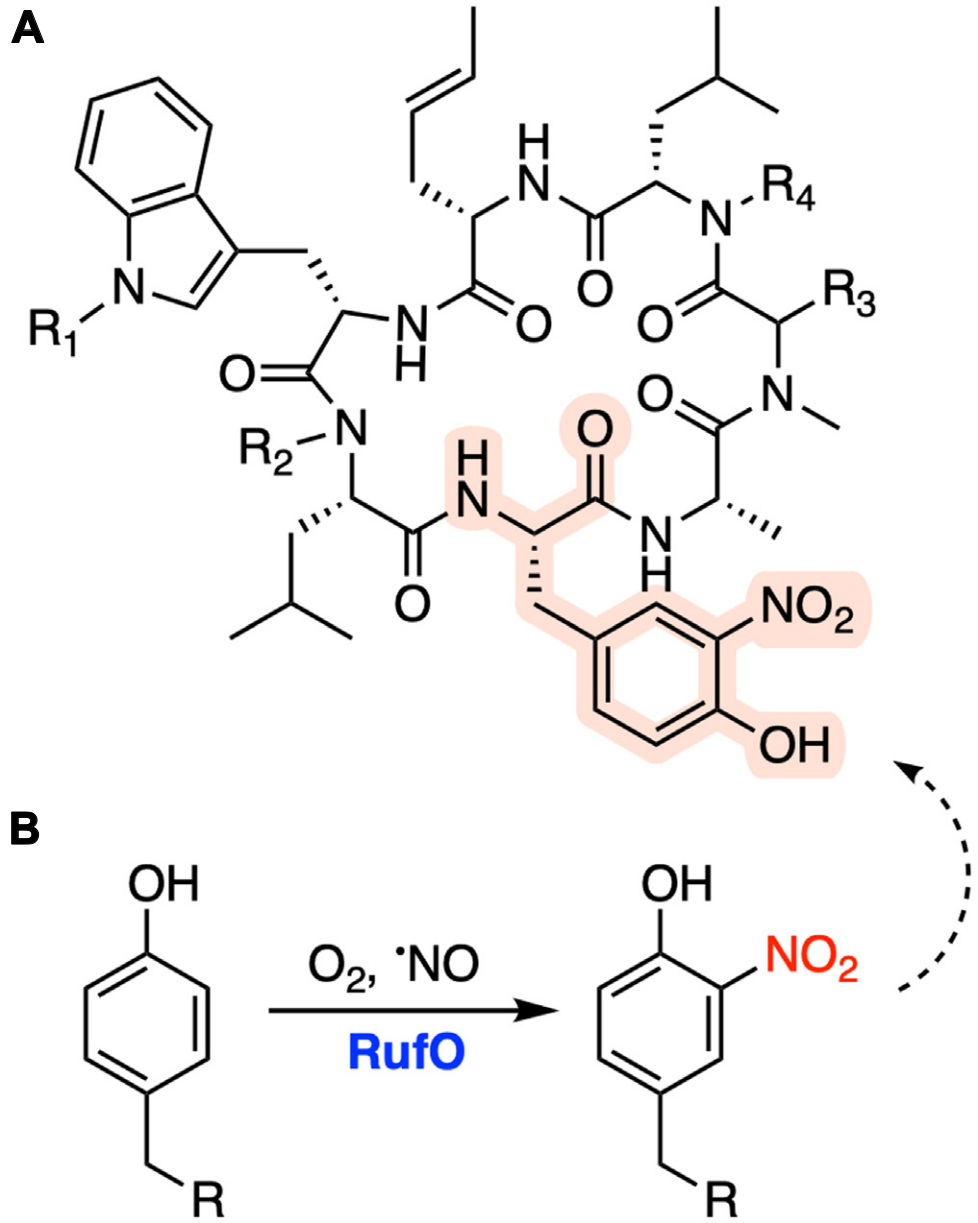
**RufO is invovled in the biosynthesis of rufomycins.***A*, general structure of rufomycins. 3-NO_2_-Tyr is highlighted. Positions R_1_-R_4_ are widely modified in rufomycins. *B*, nitration reaction catalyzed by RufO. R is an amino acid moiety proposed by a previous study (11).

RufO is classified as a cytochrome P450 (CYP)-type heme enzyme due to the observation that the binding of CO gas caused the shift of the heme Soret band to 450 nm (11). As one of the most well-characterized enzyme superfamilies, CYPs promote a wide spectrum of reactions that can be rationalized in a paradigm involving powerful ferryl intermediates (Compound I and II) (14, 15). O_2_ is the primary oxidant for the vast majority of CYPs, and the binding of NO either reversibly or irreversibly inhibits CYPs. One exception, however, is a nitric oxide reductase involved in fungal denitrification, which employs NADH as an electron donor under anaerobic conditions (16). The concurrence of O_2_ and NO usually produces nitrate and nitrite and denatures proteins. In the presence of transition metals such as heme, they could also form reactive nitrative species that irreparably damage the protein structures and functions (17, 18). Hence, RufO catalyzes an impressive reaction in which it mediates two incompatible diatomic molecules as the co-substrates to functionalize the primary substrate. A few flavohemoglobins in bacteria and yeast can subsequentially activate O_2_ and NO as denitrosylases or NO oxygenases, but neither involves catalysis of an organic substrate (19, 20). The only other reported direct nitration in nature is in the biosynthetic pathway of thaxtomin, where tryptophan (Trp) is transformed into 4-nitro-tryptophan by the enzyme TxtE (21). TxtE is much better characterized than RufO with crystal structures at atomic resolutions and established early steps in the catalytic cycle (22, 23, 24, 25, 26). Surprisingly, TxtE only shares 26% protein sequence identity with RufO (11), which is even lower than the sequence identity when compared with other CYPs. The amino acid divergence presumably suggests that RufO and TxtE play different roles in the biosynthetic pathways apart from modifying distinct substrates. In-depth biochemical characterization of RufO will add another example to nitrating metalloenzymes and expand the knowledge of heme chemistry.

The biocatalytic nitration by RufO is unusual, but tyrosyl nitration is commonly found in protein molecules in mitochondria where reactive oxidative species, such as superoxide anion, are constantly generated (27). Nitration dramatically tunes the phenolic p*K*_a_, redox potential, hydrophobicity, and volume of Tyr residues, resulting in profound structural and functional changes in proteins. For example, the nitration of several conserved Tyr residues in cytochrome *c* furnishes the protein with a new peroxidase activity but diminishes its native function in electron transport (28). Probing the structural features and substrate specificity of RufO could pave the way to design enzymatic tools for posttranslational nitration on proteins. In addition, nitroaromatics are universal building blocks in a variety of industrial chemicals, including pharmaceuticals, pesticides, dyes, and explosives (29). The conventional nitration methods of utilizing electrophilic substitution using nitric acid can be problematic, as the reaction suffers from poor selectivity and poses safety and environmental concerns. Advanced nitration methods have been recently developed, but most of them are still not suitable for regioselective or large-scale production and are limited by substrates (30). It is of interest to investigate how nitrating enzymes selectively install nitro groups, as this knowledge will aid in engineering them into biocatalysts for desired nitration under ambient conditions. Understanding the biological function of RufO is the very first step to unleashing the nitrating power to resolve these synthetic challenges.

Here, we report a biochemical and biophysical investigation of a recombinant RufO enzyme originally found in *Streptomyces atratus*, an early-established organism that produces multiple rufomycins. Its enzymatic activity and redox potentials were assessed. The heme center and its response to the addition of Tyr were characterized by absorption, electron paramagnetic resonance (EPR), and resonance Raman (rR) spectroscopic methods. In addition, a crystal structure at a resolution of 1.89 Å reveals unique structural features distinct from TxtE. We also performed the molecular docking of Tyr and a rufomycin precursor onto RufO. This work excludes the possibility of Tyr being the native substrate and points to a new biological function for RufO in the biosynthesis of rufomycin.

## Results

### Examining reduction potentials and the nitrating activity of RufO

**Figure 2:**
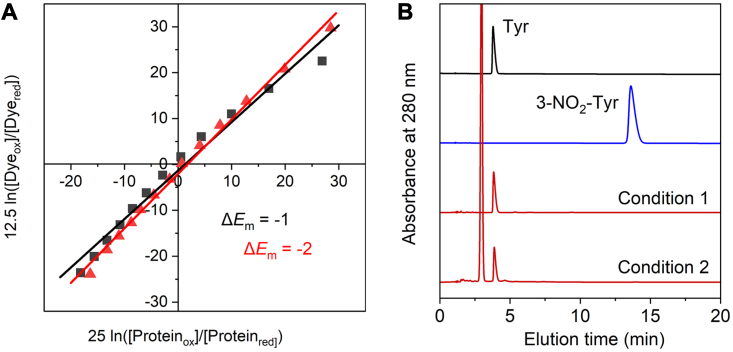
**Measurements of reduction potential and activity of RufO.***A*, Nernst plot of RufO enzyme alone (*black*) and enzyme with 1 mM Tyr (*red*). The data points are derived from the absorption spectra upon reduction shown in Fig. S2. Linear fittings yielded *y*-intercepts (Δ*E*_m_ = *E*_RufO_ -*E*_dye_) of −1 and −2, respectively. *B*, HPLC profile of RufO activity assay. The traces from top to bottom are HPLC analysis of 1 mM Tyr standard, 1 mM 3-NO_2_-Tyr standard, the reaction under condition 1 using chemically reduced enzyme, and the reaction under condition 2 using PdR/PdX redox pair. Peaks eluted around 3, 4, and 13 min are excess NADH, Tyr, and 3-NO_2_-Tyr, respectively. PdR, putidareductase; PdX, putidaredoxin.

The recombinant RufO with an *N*-terminal cleavable His_6_-tag was isolated as a red soluble protein with a molecular weight of 45.6 kDa (Fig. S1). The isolated protein was subjected to reduction potential measurements and activity tests. CYPs are known to form a redox potential gradient with various components, which allows electrons to sequentially flow to CYPs to activate oxygen with two reducing equivalents in each turnover. In bacteria, such a redox gradient can be achieved by transferring electrons from NADH (−320 mV) to a reductase containing FAD (−290 mV), then to an iron-sulfur redoxin (−240 mV for putidaredoxin [PdX]), and eventually to a CYP (31). Most bacterial CYPs show quite negative redox potentials in the resting state to avoid autooxidation, but a dramatic increase in redox potential is expected to enable the regulated multicomponent reduction. For example, the redox potential of CYP101 increases from −303 mV to −173 mV with substrate bound (31). Such a significant increase is essentially due to a coordination change when the substrate binds to the distal site. It is known that O_2_ binds prior to NO in TxtE when the enzyme is reduced for catalysis (23); hence, we were curious if the early steps in the catalytic cycle of CYPs, such as a substrate binding-triggered redox change, also apply to RufO. This prompted the utilization of a dye-coupled method to determine the reduction potentials of RufO from Nernst plots (32, 33). Absorption spectroscopic changes of RufO and the dye neutral red (*E*_*dye*_ = −325 mV) were monitored during a gradual reduction (Fig. S2). As plotted in Figure 2*A*, the reduction potentials of RufO alone and RufO with excess Tyr are −326 and −327 mV, respectively. The negligible change shown in the reduction potential of RufO suggests that the addition of Tyr barely evoked any changes in the heme center. RufO alone showed a relatively low reduction potential when compared with common CYP redox partners, meaning substrate binding is necessary for an effective reduction, as seen in most CYPs. However, Tyr is unable to induce the conversion to a more reductive heme center which is opposite to what has been known about CYPs.

The enzymatic reactions were subsequently set up under two conditions. Kinetic studies on TxtE have shown that nitration requires an external electron donor and only one reducing equivalent is needed, distinct from the two equivalents in classic CYP reactions (23, 24). The first condition chemically prereduced RufO but only allowed one reaction turnover due to the absence of a sustainable electron source. The method was successfully used in TxtE to examine the catalytic activity (23, 24). The second condition was adapted from the reported methods tested in both TxtE and RufO (11, 21, 22, 26), which involves a redox pair, putidareductase (PdR)/putidaredoxin (PdX), for multiple turnovers. There is no gene encoding a reductase or a redoxin in the reported *ruf* gene cluster (11). Also, it is unlikely for an enzyme in a bacterial secondary metabolic pathway to couple with an exclusive redox pair. Therefore, common bacterial redox pairs, such as PdR/PdX and spinach ferredoxin/reductase, should satisfy the reducing requirements for RufO, as has previously been demonstrated through studies on TxtE (21, 22, 26). Surprisingly, neither method resulted in a detectable peak of 3-NO_2_-Tyr as shown in HPLC (Fig. 2*B*). Also, no product peaks corresponding to oxygenated products, such as 3,4-dihydroxyphenylalanine as one would expect for a CYP activity, were identified. We also tried different concentrations and adding orders of O_2_, NO, and Tyr but did not observe any reaction product. We set up the enzymatic reaction of TxtE using PdR/PdX and observed the expected 4-nitro-tryptophan product (Fig. S3), which suggested that our assay method was robust. A trace of the product was observed in the previous RufO study (11), which could result from nonenzymatic reactions in which transition metals, such as heme, activate O_2_ and NO to generate reactive nitrative species that are capable of tyrosyl nitration (34, 35).

### Spectroscopic characterization of the heme center in the absence and presence of tyrosine

**Figure 3:**
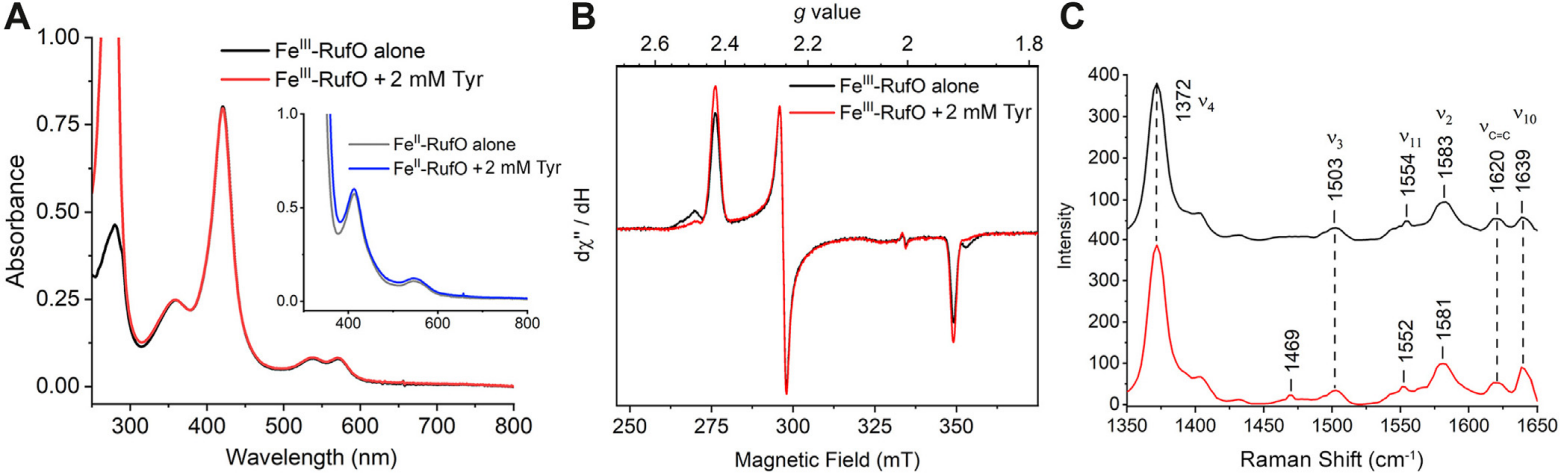
**Spectroscopic analysis of RufO heme center in the presence and absence of 2 mM Tyr.***A*, absorption spectra of oxidized RufO enzyme alone (*black*) and enzyme with 2 mM Tyr (red). Inset: spectra of reduced RufO enzyme alone (*gray*) and enzyme with 2 mM Tyr (*blue*). *B*, X-band continuous wave EPR spectra of oxidized RufO enzyme alone (*black*) and enzyme with 2 mM Tyr (*red*). The spectra were collected at 30 K with a microwave power of 1.0 mW. *C*, resonance Raman spectra of RufO enzyme alone (*black*) and enzyme with 2 mM Tyr (*red*). The spectra were collected using the 413.1 nm laser line at 5 mW. EPR, electron paramagnetic resonance.

The unchanged reduction potential of RufO and the absence of *in vitro* activity cast doubt on the finding that Tyr is the native substrate of RufO. Therefore, a series of spectroscopic tools were employed to evaluate how the heme center responds to the addition of Tyr. The oxidized RufO showed a Soret band pronounced at 421 nm and Q bands at 538 and 570 nm (Fig. 3*A*), in agreement with the reported absorption spectra of RufO and TxtE (11, 21). This result indicates that a cysteine-ligated heme was properly incorporated into the purified enzyme. Tyr was then titrated into the enzyme, but no significant changes were observed even when 2 mM Tyr was added, indicating the electronic structure of heme was not altered in the presence of excess Tyr. The first RufO study reported a type I-shift of the Soret band upon the binding of Tyr, and a binding constant (*K*_D_) of 0.1 μM was measured (11). However, the spectral difference was negligible and could result from titration artifacts. In contrast, an apparent type I-shift from 420 to 390 nm was observed in TxtE, and a *K*_D_ of ∼ 40 μM was determined by independent groups (21, 22). RufO and TxtE both were reported with low activities (11, 36), so *K*_M_ values are not expected to be dramatically different from the *K*_D_. Hence, the *K*_D_ of submicromolar concentration is not anticipated as both RufO and TxtE are enzymes in secondary metabolic pathways. A tight binding of Tyr could cause the depletion of the amino acid essential for protein synthesis and primary metabolic pathways. We also examined the binding of Tyr in the reduced state. RufO was reduced by excess sodium dithionite and mixed with Tyr under anaerobic conditions. The reduced RufO showed absorption features at 413 and 547 nm. Similarly, no obvious spectral changes were found upon the addition of 2 mM Tyr (Fig. 3*A*, inset).

UV-vis spectroscopy is less sensitive to subtle changes at the heme center; thus, EPR and resonance Raman (rR) spectroscopies were used to determine the electromagnetic and vibrational properties of the heme. X-band continuous wave EPR spectrum of the oxidized RufO showed a rhombic low-spin species (*S* = 1/2) at *g* = 2.43, 2.25, and 1.92 (Fig. 3*B*). A minor species, presumably a minor conformation or the result of freezing artifacts, was also observed at *g* = 2.48 and 1.90. The major low-spin signal is typical for CYPs due to the binding of a distal water ligand. Substrate addition is expected to displace or interfere with the water ligand and, thus, convert the low-spin heme to a high spin (*S* = 5/2) or shift the *g* values. However, the spectrum after adding 2 mM Tyr looked very similar, with the exception that the minor species was seemingly converted to the major species without changing the *g* values. We also scanned the samples at various temperatures (10 ∼ 40 K), and the high-spin region remained silent (Fig. S4). Overall, no substrate-induced changes were found in the active site, which is the opposite of most CYPs that lose or weaken the ligation of the axial water upon substrate binding. We also prepared NO-bound ferrous complexes of RufO to probe possible interactions between the heme-bound diatomic molecule and the substrate. As shown in Fig. S5, a low-spin (*S* = 1/2), six-coordinate iron-nitrosyl complex, known as a porphyrin-based {FeNO} (7), was shown, which is similar to other ferrous nitrosyl complexes formed by thiolate-ligated heme proteins (37). Minor changes were detected when 2 mM Tyr was introduced, but the *g* values and the hyperfine splitting caused by NO remained the same, suggesting that Tyr did not bind in the close vicinity to heme to interact with the nitrosyl complex or heme-based reactive intermediates.

The binding of Tyr was also probed by rR spectroscopy. In the high-frequency region, RufO enzyme alone and the enzyme with excess Tyr showed generally similar spectra (Fig. 3*C*). The oxidation marker band ν_4_ appeared at 1372 cm^−1^ for both samples, which is expected for the ferric oxidation state. The core size marker bands, ν_3_, ν_2_, and ν_10_, are sensitive to spin state and coordination number; therefore, they are expected to change upon substrate binding. However, ν_3_ and ν_10_ did not shift, and only trivial shifts (less than 2 cm^−1^) were shown for ν_2_ when 2 mM Tyr was added, inferring a six-coordinate low-spin heme that is not responsive to Tyr, which is consistent with our UV-visible and EPR results. Typically, these bands show apparent downshifts in CYPs when substrates bind, indicating a conversion from low-to high-spin due to the loss of the heme-bound water molecule (38, 39, 40). Based on literature precedent, the ν(C=C) at 1620 cm^−1^ defines the orientations of vinyl groups with respect to the pyrrole rings. One vinyl stretching mode ν(C=C) appearing at 1620 cm^−1^ suggests the presence of mainly one vinyl conformation with a torsional angle |τ| of 100−180° or 0−10° (39), which is confirmed by our crystal structure (discussed below) showing the torsion angles |τ| of 164° and 165° for the vinyl groups.

Overall, multiple independent spectroscopic methods identified that the heme center of RufO is not approachable by Tyr. All results indicated that Tyr is unable to induce a structural rearrangement in the active site of RufO that is generally expected in most CYPs. Tyr either binds far from the heme center so that only marginal perturbation can be detected, or Tyr is not the native substrate of RufO.

### A 1.89-Å crystal structure of RufO

**Figure 4:**
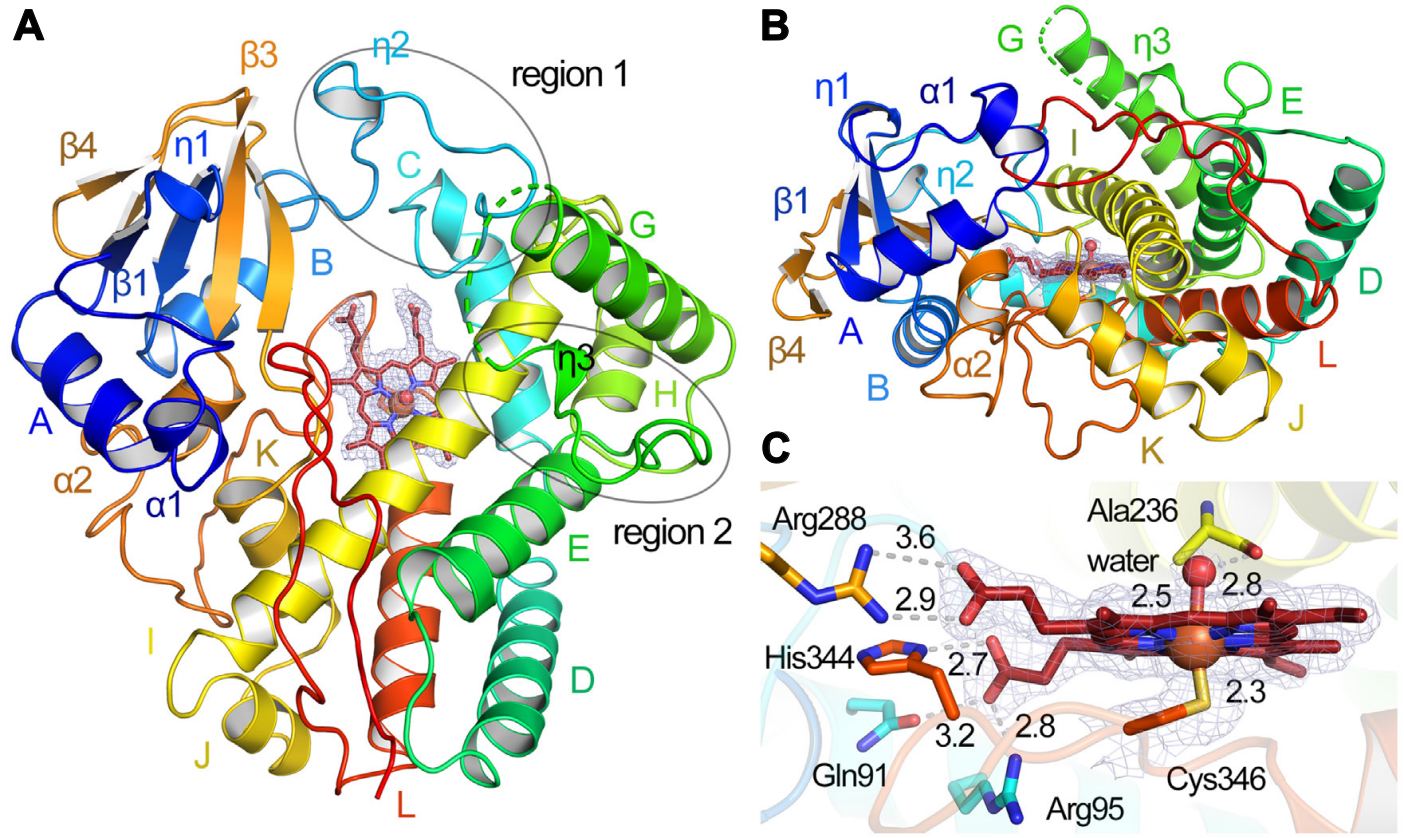
**A 1.89-Å crystal structure of RufO**. (*A*) *top* view and (*B*) side view of the overall structure and (*C*) the heme proximal site. The cartoon representation is colored by a *rainbow* spectrum from a *blue N*-terminus to a *red C*-terminus. The α-helices, β-sheets, and *3*_10_ helices are noted. The N, O, S, Fe, and heme C atoms are in *blue*, *red*, *yellow*, *brown*, and *deep red*, respectively. The 2*F*_o_–*F*_c_ difference maps of the heme center are colored in *light blue* and contoured at 1.0 σ. The *gray dashed lines* indicate distances (Å) between atoms. Regions 1 and 2 showing the largest structural discrepancies when compared with TxtE and CYPs are marked by *black circles*. CYP, cytochrome P450.

To further investigate if RufO binds Tyr, we determined a crystal structure with a resolution of 1.89 Å. Attempts to use TxtE structures as search models for molecular replacement (MR) failed, possibly due to the low similarity in protein sequences. An MR solution was found when using the structure of a mycobacterial CYP164A2 which shows 34% sequence identity with RufO (41). The RufO crystal structure belongs to the *P*4_3_2_1_2 space group, and each asymmetric unit contains one monomer, consistent with our gel filtration results in which RufO is a monomer in solution (Fig. S6). Detailed crystallographic data statistics are shown in Table S1. RufO contains thirteen α-helices (A-E, G-L, α1, and α2), three β-sheets (β1, β3, and β4), and three *3*_10_ helices (η1 to η3) (Fig. 4, *A* and *B*). α-helices and β-sheets were labeled following the nomenclature used in CYPs and TxtE (26, 42). RufO was crystallized under multiple conditions, and the structures obtained from different conditions are mostly identical. In the 1.89-Å structure, the majority of the residues were modeled based on clear electron densities, except for a disordered *N*-terminus (residues 1–10) and a region between η3 and α-helix G (residues 166–181). The region is often dynamic in CYPs and is also disordered in TxtE (22, 26, 43). The structure mostly resembles the trigonal-prism fold found in CYPs and TxtE, however, several key differences are noticed. First, β2 is conserved in most CYPs, and β5 is conserved in CYPs and TxtE, but they are missing in RufO; second, two α-helices following the B helix, annotated as B′_1_ and B′_2_ in TxtE (26), are replaced by η2 and loops in RufO; lastly, α-helix F is conserved in TxtE and CYPs but appears as η3 and loops in RufO. The latter two regions show the largest structural discrepancies when compared with the structures of TxtE and CYPs, and they are defined as regions 1 and 2 hereafter (Figs. 4*A* and S7). Superposition of structures of RufO and TxtE resulted in a root-mean-square deviation of 2.01 Å over 335 C_α_ atoms, showing that the two enzymes are considerably similar in structure (Fig. S7). However, RufO is composed of more loop regions and presents more flexibility than TxtE and most CYPs.

**Figure 5:**
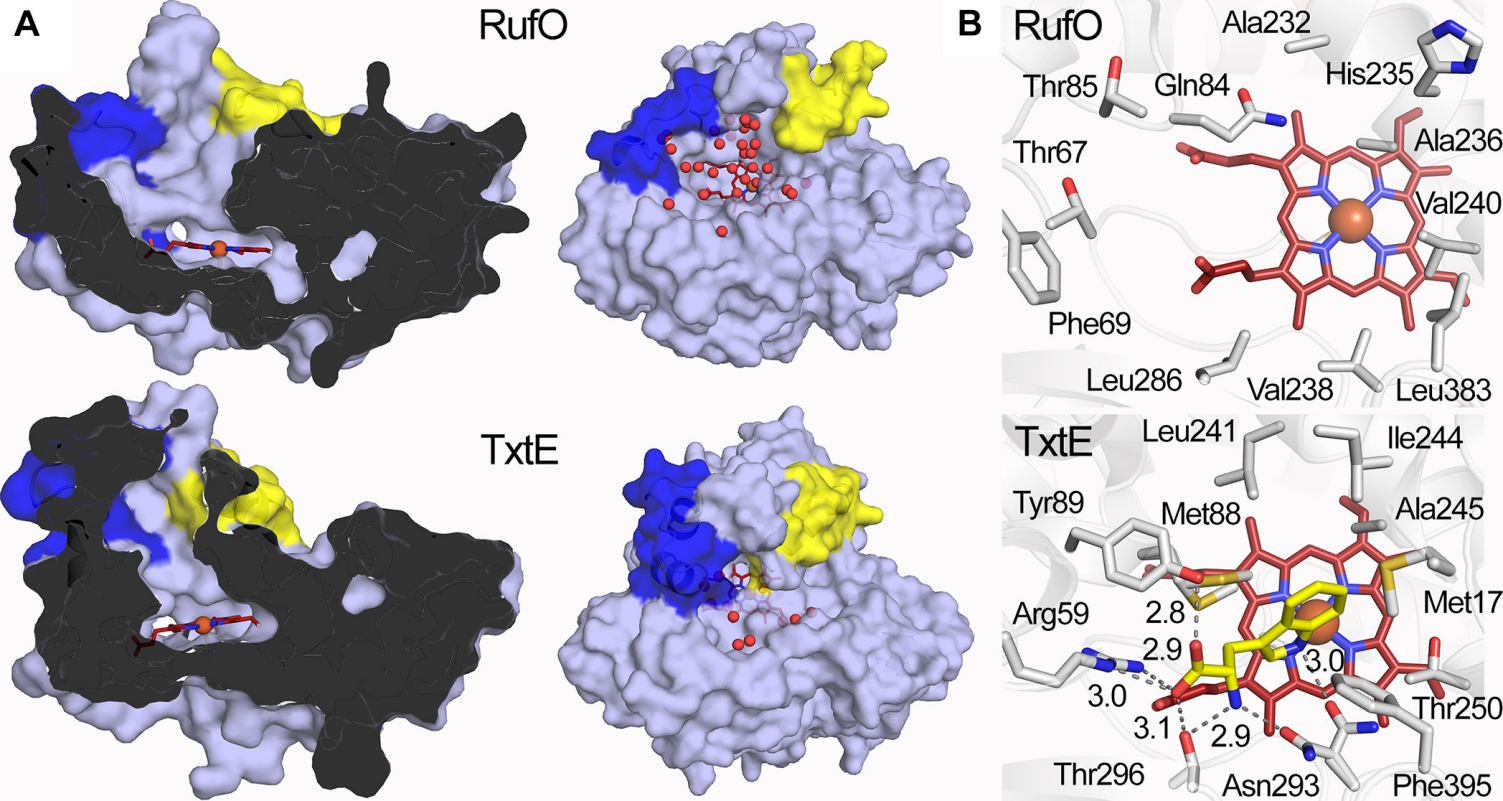
**Comparison of RufO and TxtE structures.***A*, vertical cross sections (*left*) and top views (*right*) of RufO (*top*) and TxtE (*bottom*) in surface representation. Regions 1 and 2 are colored in *blue* and *yellow*, respectively. Water molecules in the substrate access channels are shown in *red spheres* in the top views. The PDB entry of the TxtE structure is 4L36. *B*, distal pockets of RufO (*top*) and TxtE (*bottom*). Color code of atoms: protein carbon, *white*; tryptophan carbon, *yellow*; heme carbon, *deep red*; iron, *brown*; nitrogen, *blue*; oxygen, *red*. *Gray dashed lines* indicate distances (Å) between atoms. Active site water molecules are removed for clarity. The PDB entry of the tryptophan-bound TxtE structure is 4TPO. PDB, Protein Data Bank.

Regions 1 and 2 are located on the protein surface, guarding the entrance of the heme distal pocket, which is anticipated to dictate substrate selectivity and binding. Indeed, a single mutation in the loop connecting helix F of region 2 in TxtE completely shifts the regioselectivity of the enzyme (43). A ligand-free TxtE structure is available, but it has unresolved electron densities at the helix B′_1_, which alters the channel shape. Hence, the TxtE structure here used for comparison is an imidazole-bound structure with a resolved B′_1_ conformation at a comparable resolution (2.10 Å) (26). As shown in Figure 5*A*, regions 1 and 2 gate substrate access channels in RufO and TxtE. RufO has a larger substrate access channel than TxtE due to the widening of these two regions. As a result, the opened active site in RufO is filled with water molecules, and the heme is largely exposed to solvent. In contrast, the TxtE structure shows the enzyme has a buried heme center with much fewer solvent molecules in the access channel, which is common in CYPs where the heme is well-protected deep inside the proteins. The differences presumably rationalize why hydrophobic Tyr is less likely to be stabilized by the solvent-accessible active site of RufO, while Trp, also a hydrophobic but bulkier amino acid, is readily accommodated in the more closed and hydrophobic active site of TxtE. Under specific conformational changes, for example, upon protein-protein interactions, the flexible loops and the short *3*_10_ helices in regions 1 and 2 could potentially close up the distal pocket and increase the hydrophobicity by releasing water molecules, which is expected to facilitate the substrate binding. Interestingly, the CYP164A2 structure, used as the search model for MR, is also reported to have an enlarged and adaptive active site that could accommodate a range of bulky substrates and inhibitors (41). Overall, our crystal structure disfavors the binding of Tyr. Instead, the active site of RufO is seemingly designed for a sizable substrate, and a significant structural rearrangement is necessary for desolvation or thermodynamic changes to stabilize the substrate.

### Active site architecture of RufO

Cys346 from the loop connecting α2 and helix L coordinates the heme from the proximal site, with an Fe-S bond distance of 2.3 Å (Fig. 4*C*). A water molecule is weakly associated with the heme, forming the sixth ligand with a distance of 2.5 Å from the iron, which is in agreement with the six-coordinate low-spin species observed in EPR and rR spectra. The water molecule is stabilized by the carbonyl group of Ala236 from α-helix I poised above the heme. The orientation of the heme is similar to those in TxtE and CYPs. In addition to the proximal axial ligand, the heme is supported by multiple residues, including Glu91, Arg95, Arg288, and His344, through salt bridge interactions with heme propionate groups (Fig. 4*C*). The Glu91 is replaced by His in TxtE and some CYPs, but other heme stabilizing residues are highly conserved (Fig. S8). In CYPs, the proximal side of heme is the binding site for the electron transfer complex (44). RufO and TxtE do not show a significant difference in residues in the proximal site, suggesting these two enzymes likely share a universal electron transfer process through the redox partners as aforementioned.

We attempted to cocrystallize RufO with Tyr and incubate the ligand-free RufO crystals in Tyr-containing solutions for an extended time. Crystals obtained under both conditions diffracted well, but unfortunately, additional electron densities corresponding to Tyr were not found in the distal pocket. This result is unsurprising since our activity assays and spectroscopic characterization showed that Tyr had difficulty binding to the active site.

A close comparison of the distal pocket residues of RufO and TxtE further explains why Tyr is not a suitable substrate for RufO. The substrate-bound structure of TxtE was obtained by incubating the crystals in Trp-containing solutions (22). As shown in Figure 5*B*, the amino acid moiety of the Trp is strongly supported by Tyr89, Arg59, Thr296, and Asn293 through hydrogen bonding and salt bridge interactions in TxtE. These residues are highly conserved in TxtE homologs, but none of them are retained in the active site of RufO. The corresponding positions in RufO are mostly nonpolar and uncharged residues, including Thr85, Thr67/Phe69, Leu286, and Val238, that are incapable of stabilizing the amino acid moiety. Furthermore, a hydrophobic border in TxtE defined by bulky nonpolar residues, including Met88, Leu241, Ile244, Ala245, Met17, and Phe395 (ordered in clockwise direction), holds the indole of Trp in place. A hydrophobic pocket is also shown in RufO but is composed of smaller nonpolar residues, including Ala232, Ala236, Val240, Leu383, Val238, and Leu286 (in clockwise direction), leaving a hydrophobic and spacious distal site. This observation excludes the possibility that Tyr binds in a conformation in which the amino acid moiety faces toward a different side. We also examined other residues near heme but did not see any suitable residues that potentially stabilize the amino acid moiety. This is consistent with our control experiment using Trp, as neither binding nor activity was observed (Figs. S9*A* and S10*A*). By comparing the active site architecture of the two nitrating enzyme, it is clear that RufO lacks the necessary structural features to bind Tyr or any free amino acids.

### Molecular docking of RufO and testing with peptide mimics

**Figure 6:**
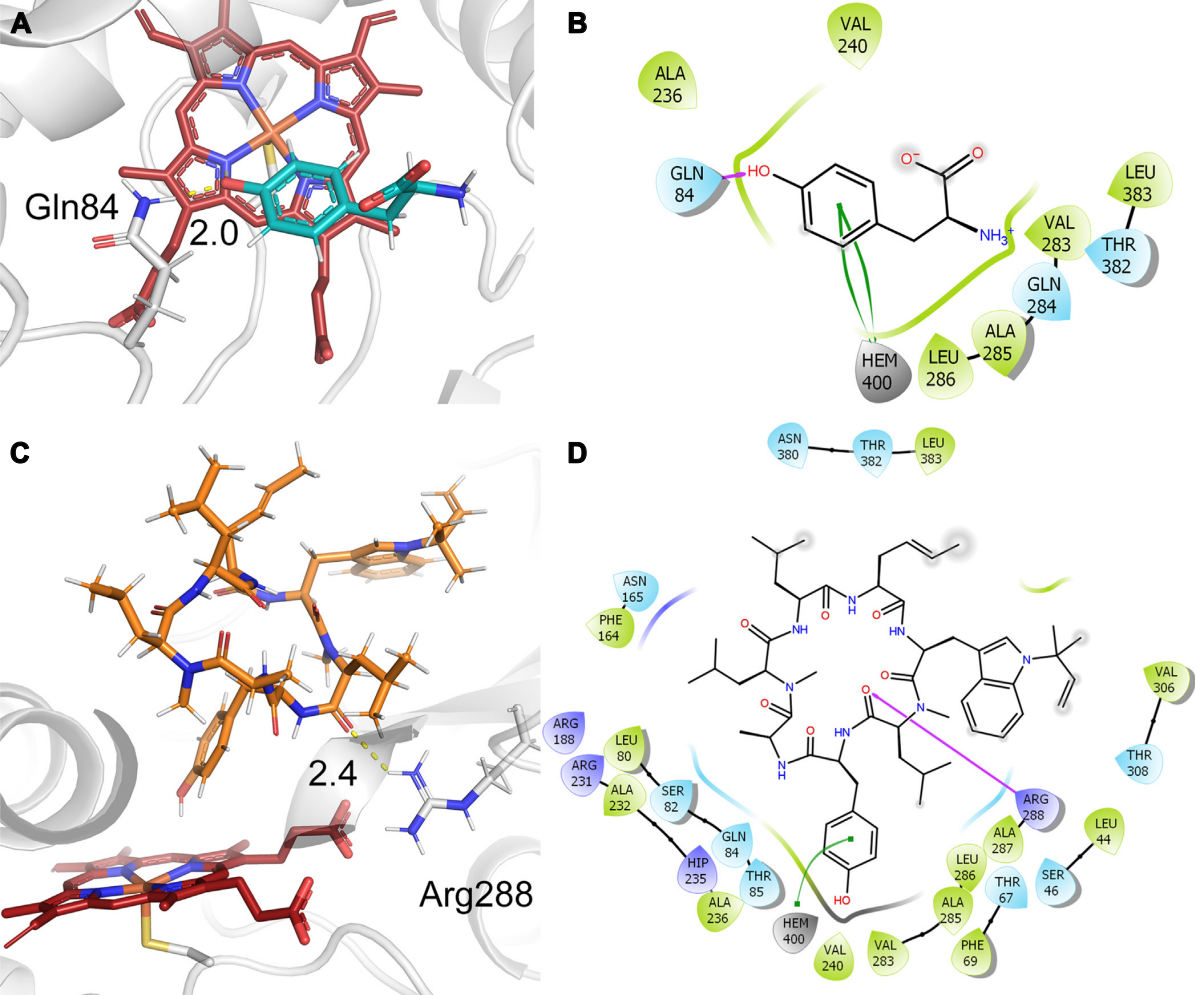
**Molecular docking of RufO.** The docking poses and 2D ligand–protein interaction diagrams for RufO docked with (*A* and *B*) tyrosine and (*C* and *D*) a cyclic peptide. Tyr, the cyclic peptide, heme, and RufO are colored in *blue*, *orange*, *red*, and *white*, respectively. In the 2D diagrams, *purple arrows* represent the hydrogen bonding; *green curves* represent the π–π stacking; *gray* shaded atoms are solvent exposed; colored droplets represent heme (*gray*), negatively charged (*red*), positively charged (*blue*), polar uncharged (*cyan*), and hydrophobic residues (*green*).

To further examine whether Tyr is a substrate of RufO, we docked a Tyr molecule onto our crystal structure. The docked pose with the best estimated binding energy of −19.61 kcal/mol (calculated with Prime molecular mechanics with generalised Born and surface area solvation panel, see Experimental procedures) is shown in Figure 6*A*. In the docking model, Tyr forms one hydrogen bond with Gln84 through the phenol group, and its aromatic ring forms π–π stacking with heme (Fig. 6*B*). However, surrounding residues do not support the amino acid moiety and the carboxylate is exposed to solvent, which is consistent with our understanding of the active site architecture. The result suggests that there is no stable binding between Tyr and RufO, and Tyr is unlikely to be the native substrate of RufO. For comparison, we also docked a Trp onto the TxtE crystal structure using the same method. The best docked pose shown in Fig. S11*A* highly resembles the previously reported crystal structure and docking models (22, 26). The estimated binding energy is −30.66 kcal/mol, much better than that of Tyr on RufO. The 2D interaction diagram shown in Fig. S11*B* highlights the interactions that are expected based on previous studies, including the hydrogen bonding between the amino acid moiety with active site residues and the π–π stacking of the indole ring with heme. Therefore, comparing the docking results of two nitrating heme enzymes, we conclude that the binding between Tyr and RufO is unstable, which agrees with our experimental data.

The widely open active site of RufO presumably binds large molecules formed during the peptide biosynthesis. Hence, we performed a docking analysis of a cyclic peptide, the rufomycin precursor formed after the cyclization by NRPS, onto our crystal structure. Fifty-seven possible docking poses were generated, suggesting the sizable active site can easily accommodate the heptapeptide with conformational flexibility. Among the 57 poses, two have the Tyr moiety bound right above the heme center and in a suitable position for nitration. The two poses showed similar binding conformations near the heme site, whereas the side chains of *N*-dimethylallyl-tryptophan and *trans*-2-crotylglycine opposite to the Tyr moiety point in different directions. Their binding energies were calculated as −43.08 and −35.81 kcal/mol, and the one with the better binding energy is shown in Figure 6, *C* and *D*. In this docking pose, the Tyr moiety forms π–π stacking interaction with heme as well as hydrophobic interactions with Ala236, Val240, and Val383. The carbonyl oxygen of an adjacent amide bond interacts with Arg288 through a hydrogen bond. Arg231 could also form a hydrogen bond with the carbonyl oxygen. Furthermore, the Leu next to Tyr in the cyclized peptide forms hydrophobic interactions with nearby residues, Thr67, Phe69, and Thr85. The detailed interactions between the peptide and RufO are shown in Fig. S12. Amino acids away from Tyr in the cyclic peptide are found with more diverse post-assembly modifications (6, 7), which is in agreement with our docking model that these residues show little interactions with RufO and are mostly solvent-exposed. Overall, the docking results not only propose a potential binding pose for bulky peptides generated in the biosynthetic pathway but also pinpoint the critical regions for the peptide binding.

The hypothesis that RufO may bind the peptides containing Tyr that has yet to be nitrated prompted an experiment to test the binding and activity of several Tyr analogs with protected amino acid moieties and peptides of varying sizes that are putatively formed after incorporating Tyr during the biosynthesis. Tyr analogs, *N*-acetyl-L-Tyr, L-Tyr methyl ester, and *N*-acetyl-L-Tyr ethyl ester, were chosen as they potentially imitate the structural characteristics of peptides that are missing in free Tyr. However, RufO was unable to bind these Tyr analogs as no shift in the Soret and Q bands was shown in absorption spectra, and no nitration activity was observed in HPLC (Figs. S9 and S10). In the previously proposed assembly sequence of amino acids, RufT starts with the modified Trp, and 3-NO_2_-Tyr is incorporated as the third amino acid. Hence, we also designed three intermediary peptides, tri-, tetra-, and pentapeptides, WLY, WLYA, and WLYAL, respectively, for binding and activity tests. Unfortunately, the tested peptide mimics showed neither binding to RufO nor product formation (Figs. S13 and S14). The negative results of the intermediary peptides suggest the possibility that a more complete cyclic peptide is preferred or the presence of RufT functional domains is required for proper substrate binding.

## Discussion

The incorporation of noncanonical amino acids is a common but essential link in the biosynthetic line of peptide-based natural products, which enhances the effectiveness of the secondary metabolites regarding interspecies defense or attraction. The mechanisms of how unique functional groups are installed, however, are not always understood. In this work, we investigated a nitrating heme enzyme found in the biosynthetic pathways of rufomycins. Interestingly, the spectroscopic and structural evidence does not support the previous substrate assignment and points to a different enzymatic role of RufO. The identity of the native substrate remains uncertain and is beyond the scope of this study. Based on the structural information, we envision a hydrophobic and bulky substrate for RufO, and substrate recognition is predicted through a large area of interactions in the access channel or at the protein surface, since residues in the close vicinity to heme are unable to provide specific interactions. It is conceivable that RufO shows substrate promiscuity due to a spacious active site and an adjustable substrate access channel.

The identified *ruf* gene cluster contains 20 open reading frames (ORFs) with respective predicted functions (11). Four CYPs encoded by four of the ORFs are the most feasible for catalyzing the nitration reaction after a careful evaluation. These four CYPs are RufC, RufM, RufO, and RufS. RufM and RufS have already been elucidated to function in regioselective epoxidation and alkyl oxidation of the cyclized peptide precursors (12). A homolog of RufC, IlaD, which shares 98% protein sequence identity with RufO, has been characterized to perform an α-oxidation in the formation of a *trans*-crotylglycine (13), another conserved nonproteinogenic amino acid in rufomycins (Fig. 1*A*). Hence, RufO is the only possible enzyme responsible for the installation of the nitro group. This notion is also supported by the first RufO study that no close matches were found when using RufO as a query for BLAST search (11), suggesting that RufO catalyzes an extraordinary reaction distinct from known CYPs. Additionally, there is always a gene encoding a nitric oxide synthetase directly next to the *rufO* homolog in every identified biosynthetic pathway of rufomycins from different organisms (11, 13). Together with our findings, the most reasonable explanation for the absence of RufO activity is that free Tyr is not the native substrate of RufO, although it could serve as a poor alternative under certain circumstances.

Many noncanonical amino acids in nonribosomal peptides are constructed prior to the selection of amino acids by NRPSs. However, modifications on natural amino acids by tailoring enzymes after forming aminoacyl NRPS domains have also been demonstrated for some cyclic peptides. For example, a CYP named P450_sky_ β-hydroxylates three NRPS-tethered amino acids in the process of the skyllamycin assembly, and another CYP named NikQ in nikkomycin biosynthesis β-hydroxylates NRPS-tethered histidine (45, 46). In these cases, the selection mechanism is independent of the amino acids but is responsive to the respective peptidyl carrier domains. Another class of transformations is targeted on NRPS-tethered intermediary peptides, which is exemplified by CYP enzymes, OxyA-C, that promote phenol coupling in glycopeptide biosynthesis (47). Most of the structural features, such as the flexible surface regions, a large substrate access channel, a solvent-accessible heme center, and hydrophobic active site architecture, identified in RufO are also found in the crystal structures of these enzymes (45, 48, 49). Therefore, RufO could represent one of these tailoring enzymes rather than a pre-assembly enzyme shunting flux of a primary metabolite. Our docking study showed the RufO cavity is large enough to bind the cyclized peptide, implying that RufO can accommodate any Tyr-containing intermediary peptides, while the presence of NRPS functional domains seems to be necessary as the intermediary peptides alone are not recognized by RufO. This idea is also supported by the isolation of a few rufomycin analogs which contain a non-nitrated Tyr (7).

The original RufO study reported that the disruption of the *rufO* gene abolished rufomycin production, but the activity was restored when 3-NO_2_-Tyr was added to the culture medium of the disruptant (11). The observation naturally led to the interpretation that the nitration of Tyr is catalyzed by RufO prior to the peptide assembly. Similarly, an independent work characterizing the production line of ilamycins from a marine *S. atratus* came to the same conclusion through a precursor feeding experiment (13). However, it is unclear how much 3-NO_2_-Tyr was supplied to the cell culture in both studies. An excess of 3-NO_2_-Tyr could force the adenylation domain of the NRPS RufT to choose 3-NO_2_-Tyr over Tyr, especially if the adenylation domain is a poor “gatekeeper” as found in some NRPSs (50). In contrast, a specificity code can be achieved by condensation domains which display high specificity toward either donor or acceptor substates, or both (51, 52). Such a case could explain the suspended biosynthesis of rufomycin in the Δ*rufO* mutant.

In summary, our study calls to attention the need to re-evaluate the timing of Tyr nitration. RufO offers great potential for introducing nitrated building blocks into nonribosomal peptide synthesis, and our study allows for the opportunity to identify the substrate selectivity and expand the substrate scope of RufO, which will provide important insights into nitrating mechanisms and enrich the knowledge of modifying enzymes in natural product biosynthesis.

## Experimental procedures

### Protein expression and purification

The codon-optimized gene of RufO from *S. atratus* was cloned into a pET28a-Tobacco Etch Virus (TEV) protease expression vector (GenScript) with a cleavable *N*-terminal His_6_-tag. To increase the solubility and stability of RufO, *Escherichia coli* BL21(DE3) competent cells (Merck) were transformed with the RufO plasmid and a pGro7 plasmid (Takara Bio) for the coexpression of GroES and GroEL chaperone proteins. The cells harboring both plasmids were cultivated in 100 ml LB broth containing kanamycin (50 μg/ml) and chloramphenicol (25 μg/ml) at 37 °C at 200 rpm. After overnight incubation (∼16 h), 5 ml of cells were added to 500 ml of LB media, along with the antibiotics of the appropriate concentrations and 3 mg/ml of arabinose. The media were incubated at 37 °C at 200 rpm until the absorbance at 600 nm reached 0.3. A freshly prepared solution of ferrous ammonium sulfate (20 μg/ml) and δ-aminolevulinic acid (40 μg/ml) was added, and the cells were allowed to continue to incubate as before until the absorbance at 600 nm reached 0.6, at which point the temperature was decreased to 28 °C and IPTG (50 μM) was added. After overnight incubation (∼16 h), the cells were harvested by centrifuging at 6000 rpm for 10 min at 4 °C and stored at −80 °C.

The frozen cells were resuspended in Buffer A (50 mM Tris–HCl and 200 mM NaCl at pH 8.0) containing β-mercaptoethanol (0.1% v/v) and phenylmethylsulfonyl fluoride (0.1 mM), with the resuspension ratio of 5 ml Buffer A per gram of cell pellet. The cells were lysed by sonication and then centrifuged to collect the supernatant. ÄKTA primer plus protein purification system (GE HealthCare) and a HisTrap FF column (Cytiva) were used to isolate RufO from the supernatant. Buffer A was used for sample loading and initial wash. A gradient of Buffer B (50 mM Tris–HCl, 200 mM NaCl, and 500 mM imidazole at pH 8.0) was applied, and RufO was eluted around 20% Buffer B. All samples and buffers were kept cold during purification. The presence and purity of RufO in eluted fractions were checked by SDS-PAGE (Fig. S1). The fractions containing pure RufO were combined and buffer exchanged (Cytiva, HiTrap Desalting column) into a desalting buffer of 100 mM Tris–HCl, 150 mM NaCl, and 5% glycerol at pH 7.5. The desalted RufO protein and TEV protease were combined and stirred at 4 °C overnight. Untagged RufO was then purified from the protein mixture by collecting the unbound fraction eluted from the HisTrap FF column. The resulting RufO samples were concentrated, aliquoted, and flash-frozen using liquid nitrogen and stored at −80 °C for future use. The protein concentration was determined by a predicted extinction coefficient of 29,450 M^−1^ cm^−1^ at 280 nm.

### Determination of reduction potentials

Reduction potentials were determined according to the method described previously with minor modifications (32, 33). Briefly, each assay was performed under anaerobic conditions and contained 300 μM xanthine (30 mM stock, Acros Organics), 14 μM RufO, 1 μM xanthine oxidase (175 μM stock, Sigma-Aldrich), and 800 μM neutral red (5 mM stock, Thermo Fisher Scientific) in a buffer of 50 mM potassium phosphate at pH 7. 1 mM Tyr was also included when determining the influence of Tyr on the reduction potential of RufO. The concentration of the dye was adjusted so that the absorbance of the dye was almost equal to that of the protein. Xanthine oxidase was added last to initiate the reduction of the protein and the dye.

Absorbance changes corresponding to the reduction of the heme and the dye were measured at 422 and 513 nm, respectively (Fig. S2). Spectra were collected at 25 °C every 2 min over a period of 60 min after the addition of xanthine oxidase on an Agilent Cary 3500 UV-Vis spectrophotometer. When spectra had remained constant for at least 10 min, excess sodium dithionite was added at the end of the reaction to obtain an absorbance reading for the fully reduced protein, after which spectra were collected until the absorbance values remained constant for at least 5 min. Data were fitted to a Nernst plot, as derived and described for a two-electron system (32, 33). The reduction potentials of RufO were calculated based on the known reduction potential of neutral red. The product of RT/nF (R = the gas constant; T = absolute temperature) in the Nernst equation, which is equal to 25/n (n = number of electrons, *i.e.*, 25 for the one-electron reduction of RufO and 12.5 for the two-electron reduction of the dye), was used to plot Figure 2*A* (33). Linear fittings resulted in equations of y = 1.06x–1.35 and y = 1.18x–2.08 for RufO alone and RufO with 1 mM Tyr, respectively. All potentials are given *versus* a normal hydrogen electrode.

### Activity assays and HPLC analysis

All reactions were set in 100 mM Tris and 150 mM NaCl at pH 7.5 with a total volume of 200 μl. The first reaction condition was derived from the reported methods used in TxtE and RufO reactions (11, 21, 22, 26). The reactions contained 50 μM RufO, 20 μM PdR, 40 μM PdX, 1 mM Tyr (99%, Beantown Chemical), 1 mM NADH (97%, Thermo Fisher Scientific), and 1 mM DEA NONOate (≥98%, Cayman Chemical). PdX and PdR expression systems were gifts from Teruyuki Nagamune (Addgene), and the proteins were expressed and purified following the reported methods (53). For TxtE reactions, the only difference was that RufO was replaced by TxtE with the same concentrations. The second reaction condition was also adapted from the reported methods (23, 24). 100 μM RufO was prereduced by 200 μM sodium dithionite (Thermo Fisher Scientific) and mixed with 1 mM Tyr and 1 mM DEA NONOate under anaerobic conditions. O_2_-saturated buffer was then added to the mixture to initiate the reaction. The reactions were mixed at 300 rpm at room temperature (RT) for 45 min and then filtered using a 10-kDa molecular weight cutoff centrifugal filter (Millipore). A 10-μL portion of filtrate was injected into an InertSustain C18 column (5 μm particle size, 4.6 × 100 mm, GL Sciences Inc) with a flow rate of 1 ml/min, and subsequently analyzed by a Thermo Fisher Scientific Ultimate-3000SD HPLC rapid separation system equipped with a photodiode array detector. The chromatograms were recorded with wavelengths from 190 to 800 nm. HPLC profiles presented in the main text were chromatograms at 280 nm. A 20-min isocratic elution method was implemented using 1.5% acetonitrile and 0.1% formic acid.

### Spectroscopic measurements

The untagged RufO samples subjected to spectroscopic measurements were prepared in the desalting buffer, in the absence or presence of 2 mM Tyr. For UV-vis absorption spectral measurements, the samples contained a protein concentration of 10 μM, and all spectra were recorded at RT using an Agilent Cary 3500 UV-Vis spectrophotometer.

Samples for EPR spectrometry were prepared with 158 μM protein and frozen in 4 mm quartz EPR tubes by liquid nitrogen. X-band continuous-wave EPR spectra were recorded using a Bruker E560 spectrometer equipped with a cryogen-free 4 K temperature system. For the ferric samples, spectra were taken at various temperatures (10–40 K), and the spectra shown in Figure 3 were collected at 30 K. For the nitrosyl complex samples, the protein was reduced by 10 mM sodium dithionite and then exposed to excess NO liberated by DEA NONOate for 10 min before being frozen in liquid nitrogen. The entire process was performed in a glove box to prevent protein denaturation. The spectra of the nitrosyl samples shown in Fig. S5 were measured at 50 K. All samples were scanned once using the SHQE high-Q resonator (9.37 MHz frequency) at a microwave power of 1.0 mW with a field modulation of 100 kHz and an amplitude of 0.6 mT. The *g* values reported were obtained by inspection of the EPR line shape.

Samples for resonance-enhanced Raman spectrometry were prepared with 158 μM protein in NMR tubes and measured at RT. The spectra were obtained at the 413.1 nm excitation from a Kr^+^ ion laser (Coherent Innova 300C) with the NMR tubes sitting on a spinning turbine. The scattered light, collected at an angle of 90º to the incident laser beam, was focused on the 100-μm wide entrance slit of an IsoPlane SCT 320 spectrometer equipped with a 1200 grooves/mm grating (Princeton Instruments), where it was dispersed and then detected by an air-cooled PIXIS CCD detector (Princeton Instruments). A longpass filter (Thorlabs) was used to remove the laser scattering. The Raman shift was calibrated using indene (Sigma-Aldrich). The laser power was kept at 5 mW for all measurements, and a total of 3600 frames were acquired over 10 min for each spectrum. Absorption spectra were taken before and after rR measurements to ensure that no laser-induced photoreduction occurred during the measurements.

### Crystallization and structure determination

The untagged RufO protein was run through a HiLoad 16/600 Superdex 75 pg column (Cytiva) using a buffer containing 50 mM Tris–HCl and 50 mM NaCl buffer at pH 7.8, followed by a concentration of the enzyme to 40 mg/ml. Multiple crystallization conditions were found for RufO alone and RufO with 5 mM Tyr using crystal screening kits of the PEG/Ion 1 and 2 screens (Hampton), Index 1 to 96 screens (Hampton), Crystal Screen 1 and 2 screens (Hampton), and Wizard 1 and 2 screens (Rigaku). The crystal structure reported in this work resulted from a condition containing 0.2 M sodium tartrate dibasic dihydrate and 20% (w/v) PEG 3350. 2 μl protein was mixed with the crystallization solution at a 1:1 ratio using a sitting drop, vapor-diffusion method at 4 °C. Red crystals were observed and grew to the optimal size after 2 weeks. The crystals were cryoprotected with crystallization buffer containing 20% (v/v) glycerol and flash-frozen in liquid nitrogen.

Data were collected at Southeast Regional Collaborative Access Team (SER-CAT) 22-ID and 22-BM beamline at the Advanced Photon Source, Argonne National Laboratory. The reported dataset was collected at 100 K at a wavelength of 1.0 Å and processed using HKL2000 (54). The structure model was built and refined using PHENIX software (https://phenix-online.org/) packages (55), and the structure of CYP164A2 with a Protein Data Bank (PDB) entry of 3R9C was used as the search model for MR.

### Molecular docking

We performed molecular docking experiments to elucidate the interaction details of the ligand-RufO binding. The crystal structure of RufO from this study was used for molecular docking. As a positive control, the structure of TxtE (PDB code: 4TPO) was retrieved to conduct the tryptophan docking. All the protein structures were prepared by the protein preparation wizard in Maestro (12.4 version, Schrodinger) (56). The protein preparation workflow contains three steps: preprocessing, optimization, and minimization. Preprocessing includes assigning bond orders, adding hydrogen, creating zero-order bonds to metals, creating disulfide, and generating het states using Epik, and so on. (57) Optimization involves optimizing the hydrogen bond using PROPKA (58, 59). Minimization is performed by using the OPLS3e force field (60). The prepared RufO structure was then used to predict binding sites by using sitemap (61, 62). The predicted site in the distal pocket was selected for receptor grid generation. For 4TPO, the existing ligand tryptophan was used for receptor grid generation. The ligands tyrosine, tryptophan, and the cyclic peptide were prepared using OPLS3e force field in Ligprep panel in Maestro (60). Ligand–protein dockings were performed in extra-precision docking mode, and peptide docking was performed in standard-precision peptide docking mode. After docking, the generated pose-viewer files were uploaded into the Prime molecular mechanics with generalised Born and surface area solvation panel to calculate binding energy. The force field used was OPLS3e (60).

## Data availability

The crystal structure has been deposited in the RCSB Protein Data Bank with the PDB code: 8SPP. The protein sequence was previously documented in NCBI with the accession number: BBA20962. All other data are contained in the article and Supporting Information.

## Supporting information

This article contains supporting information.

## Conflict of interest

The authors declare that they have no conflicts of interest with the contents of this article.
