# Supplementary material for: Structural and spectroscopic characterization of RufO indicates a new biological role in rufomycin biosynthesis
Source: J Biol Chem. 2023 Jul 13;299(8):105049. doi: 10.1016/j.jbc.2023.105049 (PMC10424215; doi:10.1016/j.jbc.2023.105049)
Supplement: Supporting information [file mmc1.docx]

**Structural and Spectroscopic Characterization of RufO Indicates a New Biological Role in Rufomycin Biosynthesis**

Stephanie Jordan^1,†^, Bingnan Li^1,†^, Ephrahime Traore^2^, Yifei Wu^3^, Remigio Usai^1^, Aimin Liu^2^, Zhong-Ru Xie^3^, Yifan Wang^1,*^

^1^Department of Chemistry, University of Georgia, Athens, GA 30602

^2^Department of Chemistry, University of Texas at San Antonio, San Antonio, TX 78249

^3^School of Electrical and Computer Engineering, University of Georgia, Athens, GA 30602

^†^These authors contributed equally to this work.

^*^Corresponding author; email: [wang.yifan@uga.edu](mailto:wang.yifan@uga.edu); ORCID ID: [0000-0003-0378-2469](https://orcid.org/0000-0003-0378-2469).

**Table of Contents**

[Table S1. X-ray Crystallography Data Collection and Refinement Statistics 2](#_Toc138861603)

[Fig. S1. SDS-PAGE analysis of protein fractions collected from nickel affinity chromatography 3](#_Toc138861604)

[Fig. S2. Absorption spectroscopic changes of RufO and neutral red during reduction 4](#_Toc138861605)

[Fig. S3. Activity analysis of TxtE 5](#_Toc138861606)

[Fig. S4. Full-scan EPR spectra of RufO with 2 mM Tyr at various temperatures 6](#_Toc138861607)

[Fig. S5. EPR spectra of ferrous nitrosyl complexes of RufO 7](#_Toc138861608)

[Fig. S6. Gel filtration chromatogram of RufO 8](#_Toc138861609)

[Fig. S7. Superposition of RufO with TxtE 9](#_Toc138861610)

[Fig. S8. Protein sequence alignment of RufO and TxtE 10](#_Toc138861611)

[Fig. S9. Absorption spectra of RufO with Trp and Tyr analogs 11](#_Toc138861612)

[Fig. S10. RufO activity assays with Trp and Tyr analogs 12](#_Toc138861613)

[Fig. S11. Docking model of TxtE with Trp 13](#_Toc138861614)

[Fig. S12. Interactions between RufO and the cyclic heptapeptide shown in the docking model 14](#_Toc138861615)

[Fig. S13. UV-vis spectroscopic binding analysis of RufO with peptides WLY, WLYA, and WLYAL 15](#_Toc138861616)

[Fig. S14 RufO activity assays with peptides WLY, WLYA, and WLYAL 16](#_Toc138861617)

[Nucleotide sequence of the codon-optimized construct of RufO 17](#_Toc138861618)

Table S1. X-ray Crystallography Data Collection and Refinement Statistics

| **PDB code** | **8SPP** |
| --- | --- |
| **Data Collection** | |
| Space group | *P*4_3_2_1_2 |
| Cell dimensions |  |
| *a, b, c* (Å) | 77.7, 77.7, 137.1 |
| ⍺, β, ɣ (˚) | 90.0, 90.0, 90.0 |
| Resolution (Å) | 50.00−1.89  (1.92−1.89)^a^ |
| Redundancy | 17.3 (19.8) |
| *R*_merge_^b^ (%) | 15.3 (93.8) |
| *I*/*σ* | 20.4 (2.3) |
| Completeness (%) | 96.9 (91.9) |
| CC_1/2_, highest resolution shell | 0.95 |
| **Refinement** | |
| Resolution (Å) | 42.87−1.89 |
| No. of reflections | 33,346 |
| *R*_work_^c^/*R*_free_^d^ (%) | 19.23/23.58 |
| No. atoms/*B*-factors (Å^2^) |  |
| Protein | 2874/38/05 |
| Heme | 43/28.97 |
| Solvent | 335/42.66 |
| Bond lengths (Å) | 0.006 |
| Bond angles (˚) | 0.836 |
| Ramachandran analysis |  |
| Favored (%) | 97.53 |
| Allowed (%) | 2.47 |
| Outlier (%) | 0.00 |

^a^ Numbers in parentheses refer to data in the highest-resolution shell.
^b^ *R*_merge_ = Σ|*I*_h_ − 〈*I*_h_〉|/Σ*I*_h_ , where *I*_h_ is the observed intensity and 〈*I*_h_〉 is the average intensity.
^c^ *R*_work_ = Σ||*F*_o_| − *k*|*F*_c_||/Σ|*F*_o_|.
^d^ *R*_free_ is the same as *R*_obs_ for a selected subset (10%) of the reflections that was not included in prior refinement calculations.


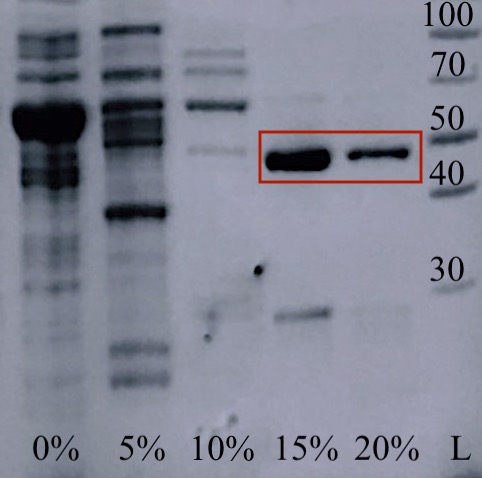


Fig. S1. SDS-PAGE analysis of protein fractions collected from nickel affinity chromatography

Lanes from left to right are fractions eluted with 0%, 5%, 10%, 15%, 20% buffer B, and PAGE ladder (L) in a 12% SDS-PAGE gel. The recombinant RufO has a molecular weight of 45.6 kDa and was shown in the 15% and 20% fractions.


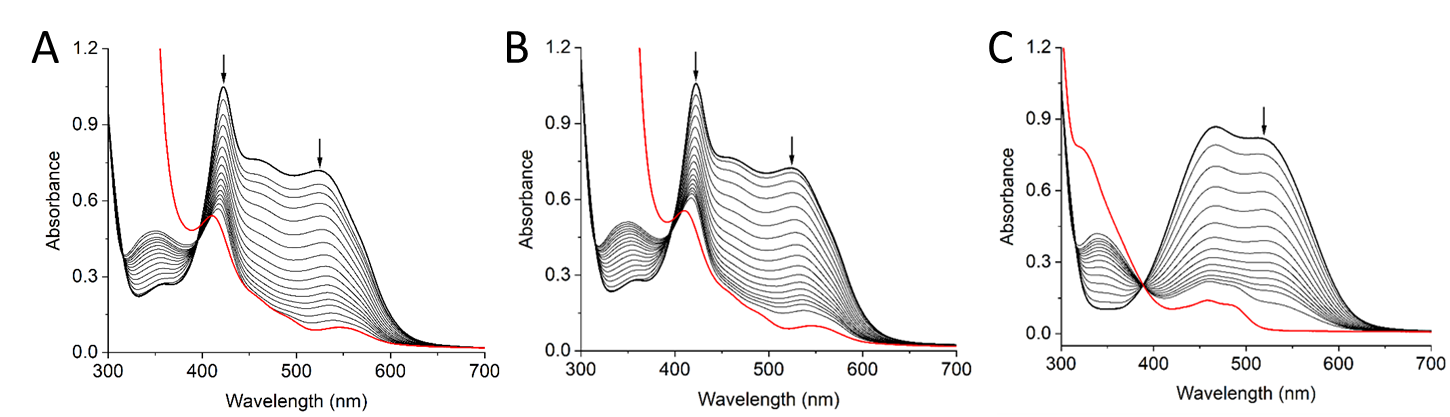


Fig. S2. Absorption spectroscopic changes of RufO and neutral red during reduction

Results were shown for (**A**) RufO and neutral red, (**B**) RufO with 1 mM Tyr and neutral red, and (**C**) neutral red alone. Upon reduction, the heme Soret bands decreased in intensity and shifted from 422 to 411 nm, and the absorption peaks of the dye also decreased. As shown in Fig. S2C, neutral red also contributes to the absorbance at 422 nm. Hence, a linear correlation of A_422_ = 0.548A_513_ – 0.067 for neutral red was considered to correct the absorbance at 422 nm when obtaining the readings of RufO from Fig. S2A and S2B.


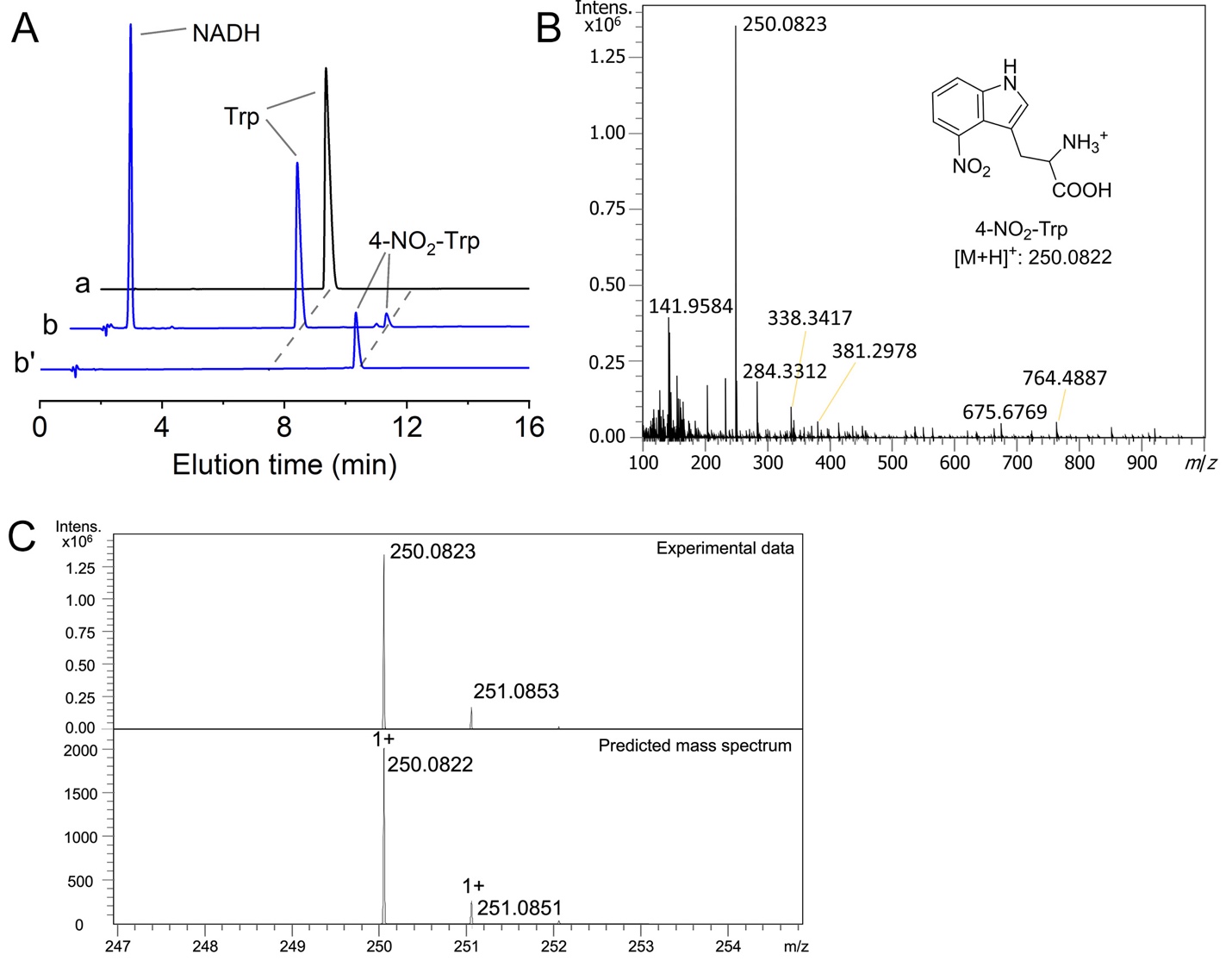


Fig. S3. Activity analysis of TxtE

(**A**) HPLC profiles of (a) 1 mM Trp standard monitored at 280 nm, (b) the reaction of TxtE monitored at 280 nm, and (b’) the same reaction monitored at 392 nm, the absorbance maxima of 4-NO_2_-Trp. The reaction was set up with 50 μM TxtE, 20 μM PdR, 40 μM PdX, 1 mM Trp, 1 mM NADH, and 1 mM DEA NONOate. (**B**) Full-range mass spectrum and (**C**) the envelope of the *m/z* = 280.0823 peak of the HPLC product fraction confirms the formation of 4-NO_2_-Trp. The mass spectrum was collected by a Bruker Impact II high-resolution mass spectrometer with an electrospray ionization source and operated in positive ion mode.

**
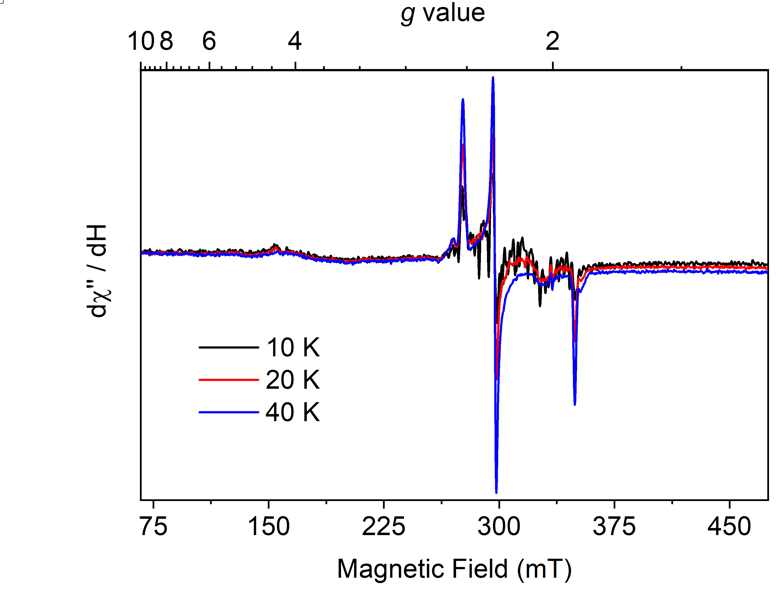
**

Fig. S4. Full-scan EPR spectra of RufO with 2 mM Tyr at various temperatures

The black, red, and blue traces were acquired at 10, 20, and 40 K, respectively. Only low-spin signals were observed at various temperatures. No spectral features or changes were observed in high-spin regions. The minimum field shown in this figure is 66.8 mT. All spectra were scanned once at a microwave power of 1.0 mW.


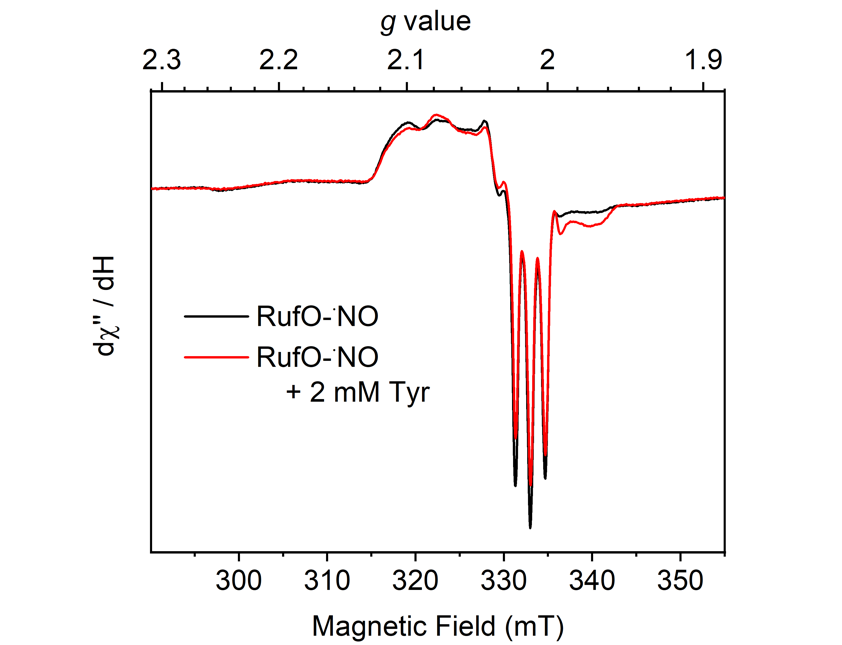


Fig. S5. EPR spectra of ferrous nitrosyl complexes of RufO

Red and black traces are samples without and with 2mM Tyr, respectively. The spectra were scanned once at 50 K with a microwave power of 1.0 mW.


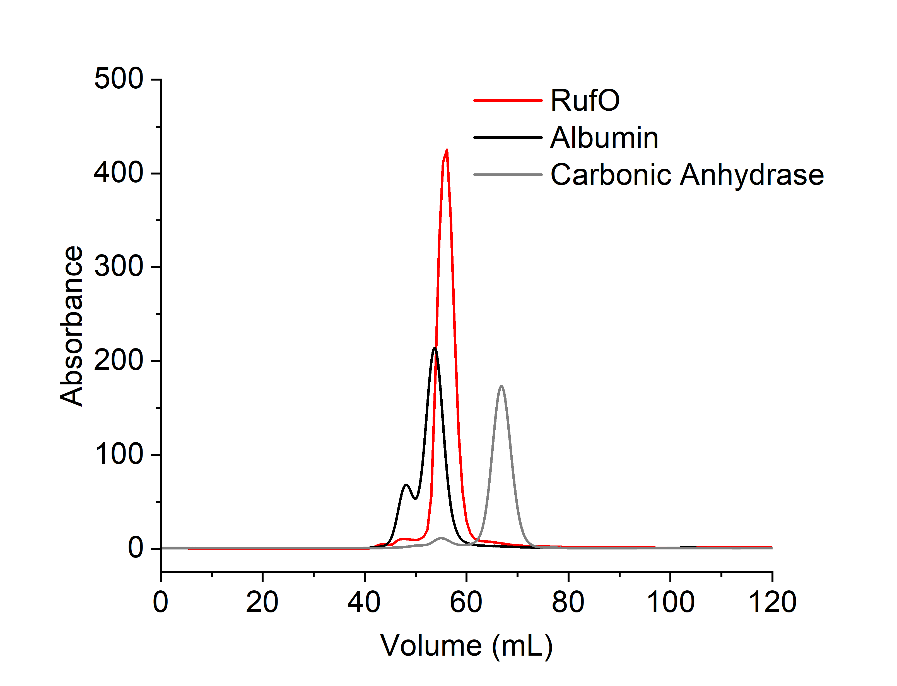


Fig. S6. Gel filtration chromatogram of RufO

RufO eluted at 55.9 mL from a HiLoad 16/600 Superdex 75 pg column with a flowrate of 1.5 ml/min using a buffer containing 50 mM Tris-HCl and 50 mM NaCl buffer at pH 7.8. Two standards, carbonic anhydrase (MW approximately 29 kDa) and bovine serum albumin (MW approximately 66 kDa), were used from the Sigma-Aldrich Gel Filtration Markers kit and eluted at 67.1 mL and 53.9 mL, respectively. The carbonic anhydrase standard and bovine serum standard were prepared with a concentration of 3 mg/mL and 10 mg/mL, respectively, and the column was injected with 1 mL of each standard. Since monomeric RufO has a calculated MW of 45 kDa, the 55.9-mL elution volume suggests that RufO is in a monomeric form in solution.


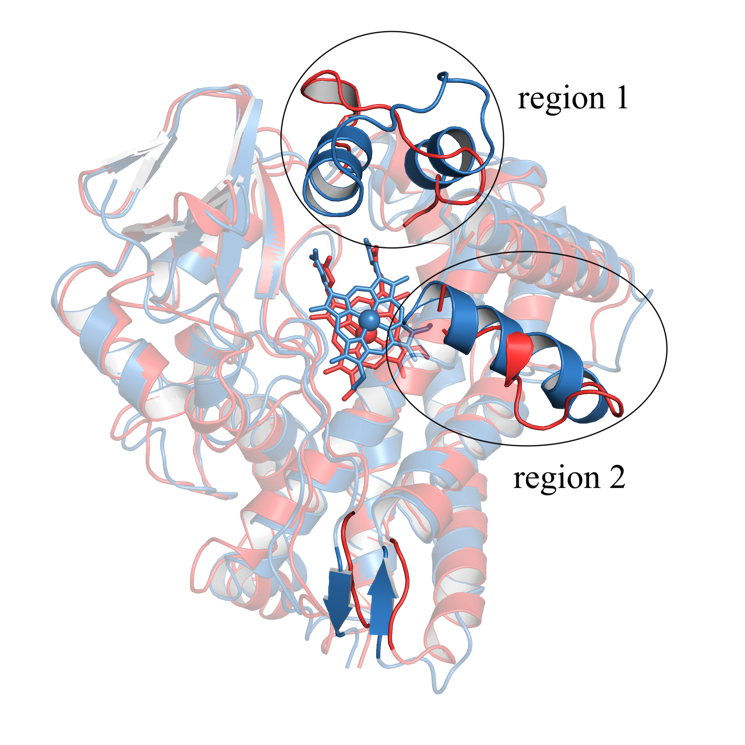


Fig. S7. Superposition of RufO with TxtE

The RufO structure reported in this work is shown in red, and the TxtE structure from the PDB entry 4L36 is shown in blue. Secondary structural elements with high similarity are shown by transparency, and key differences are highlighted with solid colors. Regions 1 and 2 showing the largest discrepancies are circled. A rmsd of 2.01 over 335 C_ɑ_ atoms was obtained.


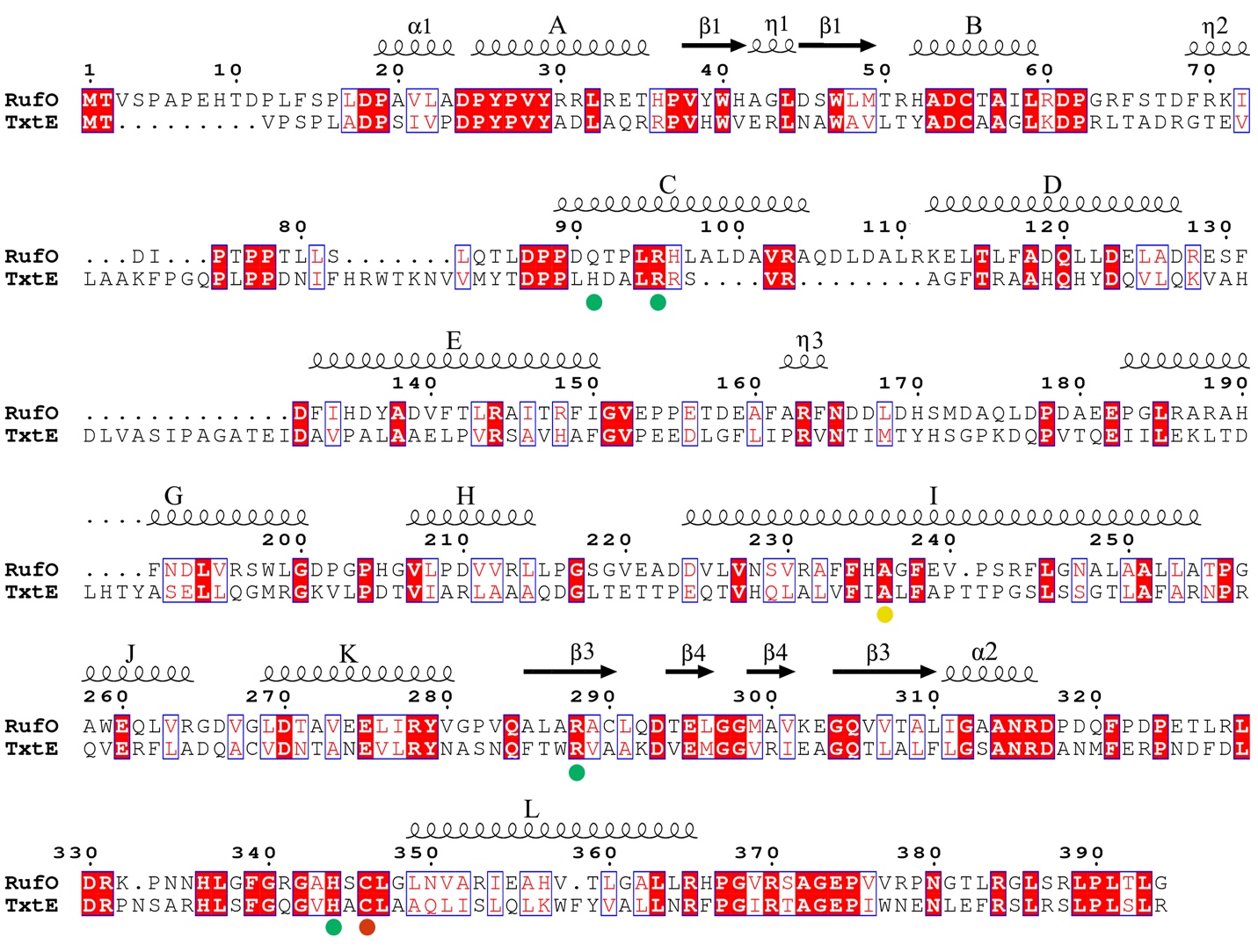


Fig. S8. Protein sequence alignment of RufO and TxtE

Strictly conserved residues are colored in white with a red background. The secondary structure elements are defined by the RufO structure reported in this work. The axial Cys ligand is marked by a red circle. The Ala forming hydrogen bonding interactions with the axial water ligand is marked by a yellow circle. The residues forming interactions with the heme propionates are marked by green circles. The sequences included are RufO from *Streptomyces atratus* (Accession number: BBA20962) and TxtE from multispecies *Streptomyces* (WP_010352784).

**
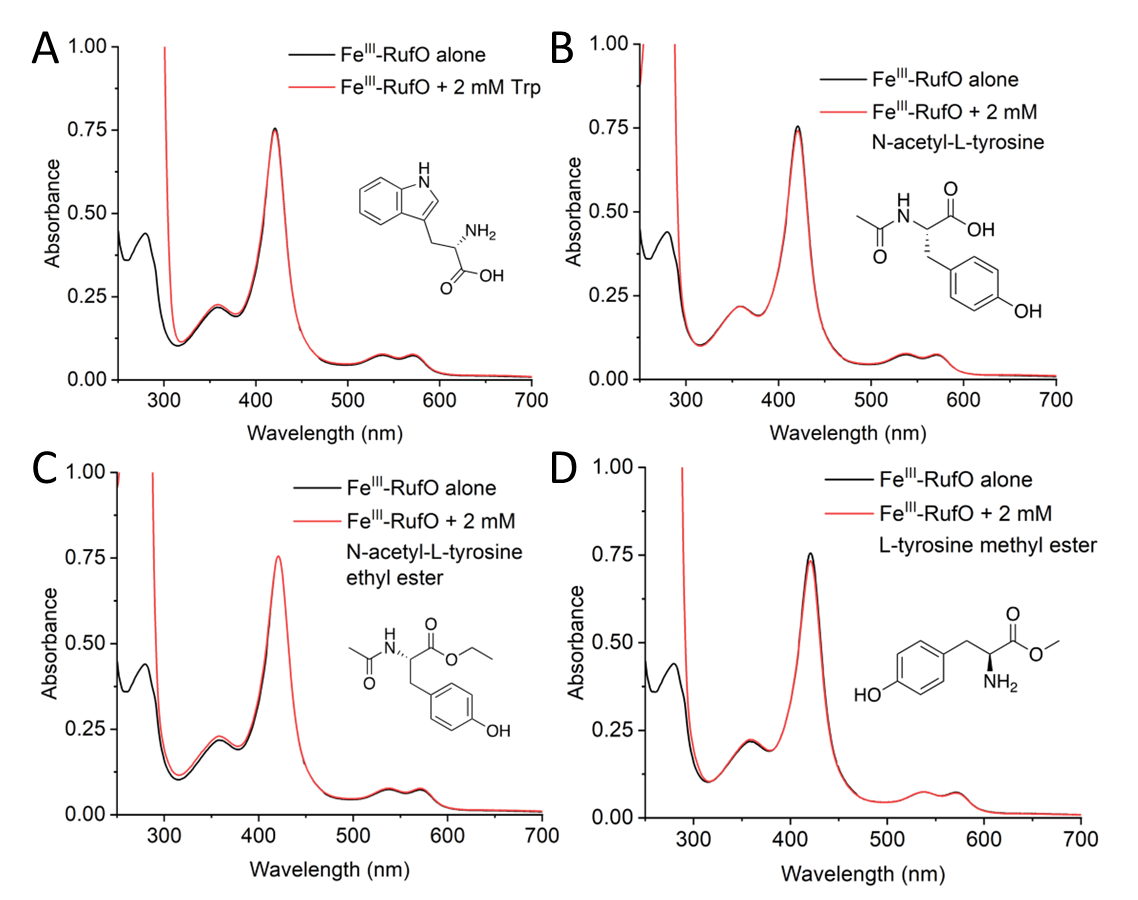
**

Fig. S9. Absorption spectra of RufO with Trp and Tyr analogs

RufO binding assays with 2 mM (**A**) Trp, (**B**) *N*-acetyl-L-tyrosine, (**C**) *N*-acetyl-L-tyrosine ethyl ester, and (**D**) L-tyrosine methyl ester. Spectra of RufO alone and RufO with the compounds are shown in black and red, respectively. For all four compounds, no shift in the Soret band was observed, indicating a lack of binding to the active site.


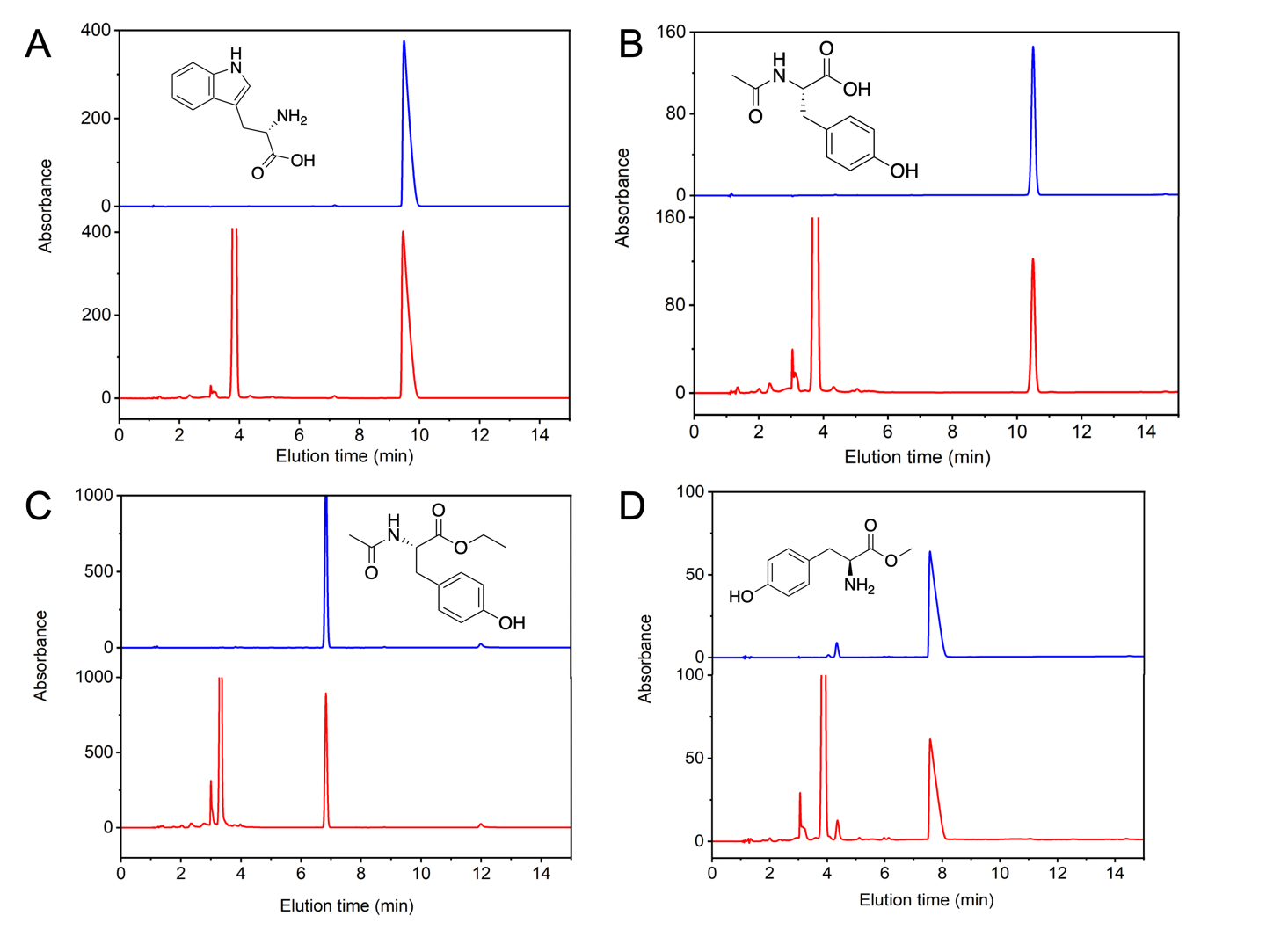


Fig. S10. RufO activity assays with Trp and Tyr analogs

HPLC analysis of RufO activity with 1 mM (**A**) Trp, (**B**) *N*-acetyl-L-tyrosine, (**C**) *N*-acetyl-L-tyrosine ethyl ester, and (**D**) L-tyrosine methyl ester. Compound standards are represented in blue and enzymatic reactions are shown in red. All traces are shown with absorbance at 280 nm. No peaks corresponding to nitrated compounds (expected to elute after the starting materials) were observed in all reactions. The peaks observed with elution times before 4 min are associated with excess NADH and derivatives. For N-acetyl Tyr ethyl ester, the mobile phase is a gradient of 0-50% acetonitrile and 0.1% formic acid in 15 min. For other compounds, the mobile phase is a gradient of 0-20% acetonitrile and 0.1% formic acid in 15 min. Other details of the HPLC method are the same as described in Experimental Procedures.


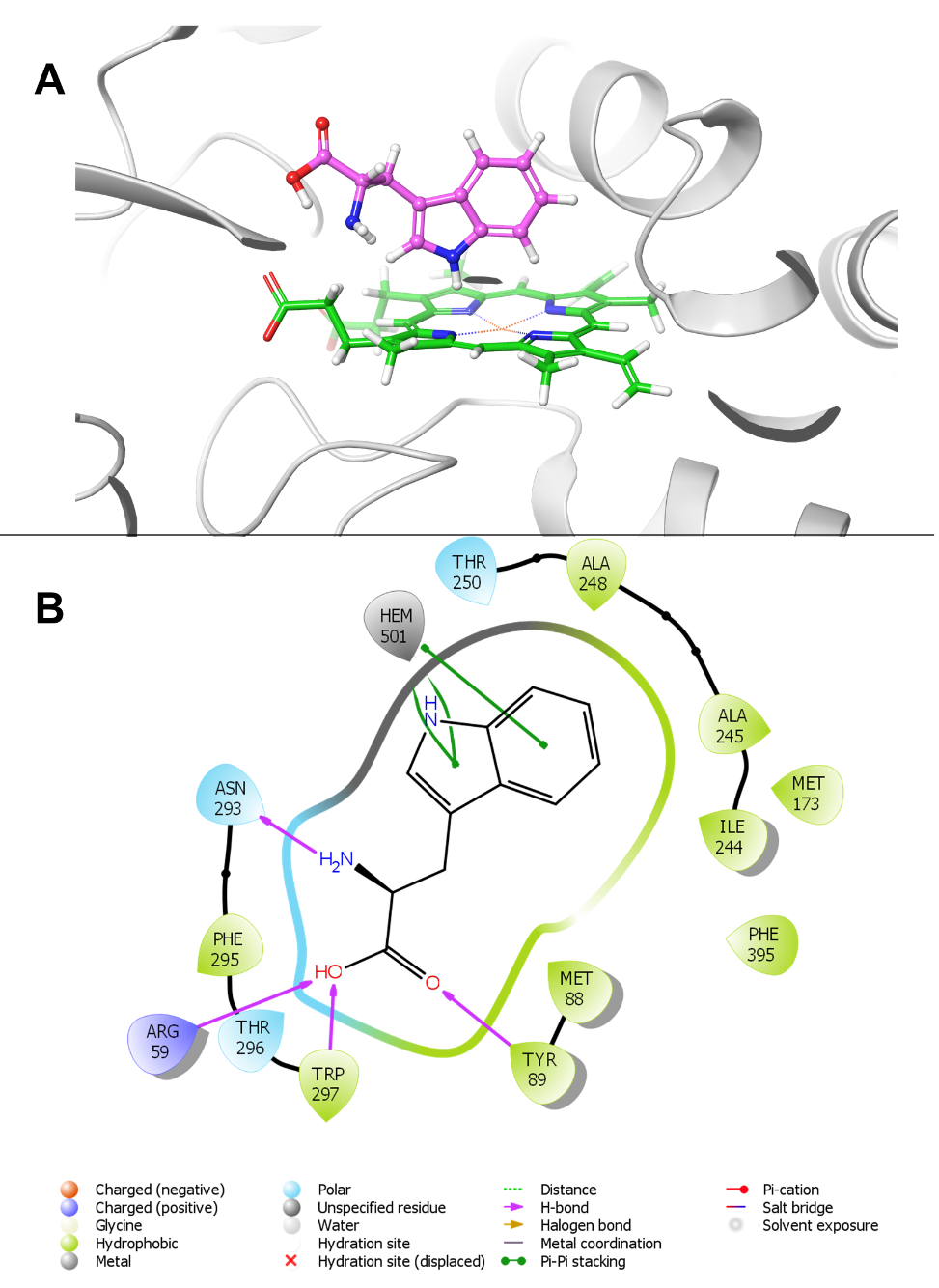


Fig. S11. Docking model of TxtE with Trp

(**A**) The docking pose with the best estimated binding affinity. Trp and heme are colored in magenta and green, respectively. (**B**) The 2D ligand–protein interaction diagram of Tyr docked onto RufO. The purple arrows represent the H-bond between the amino acid moiety of Trp and active site residues, and the green curves represent the π–π stacking between the aromatic ring of Trp and the heme. The PDB entry of 4TPO was used for docking.


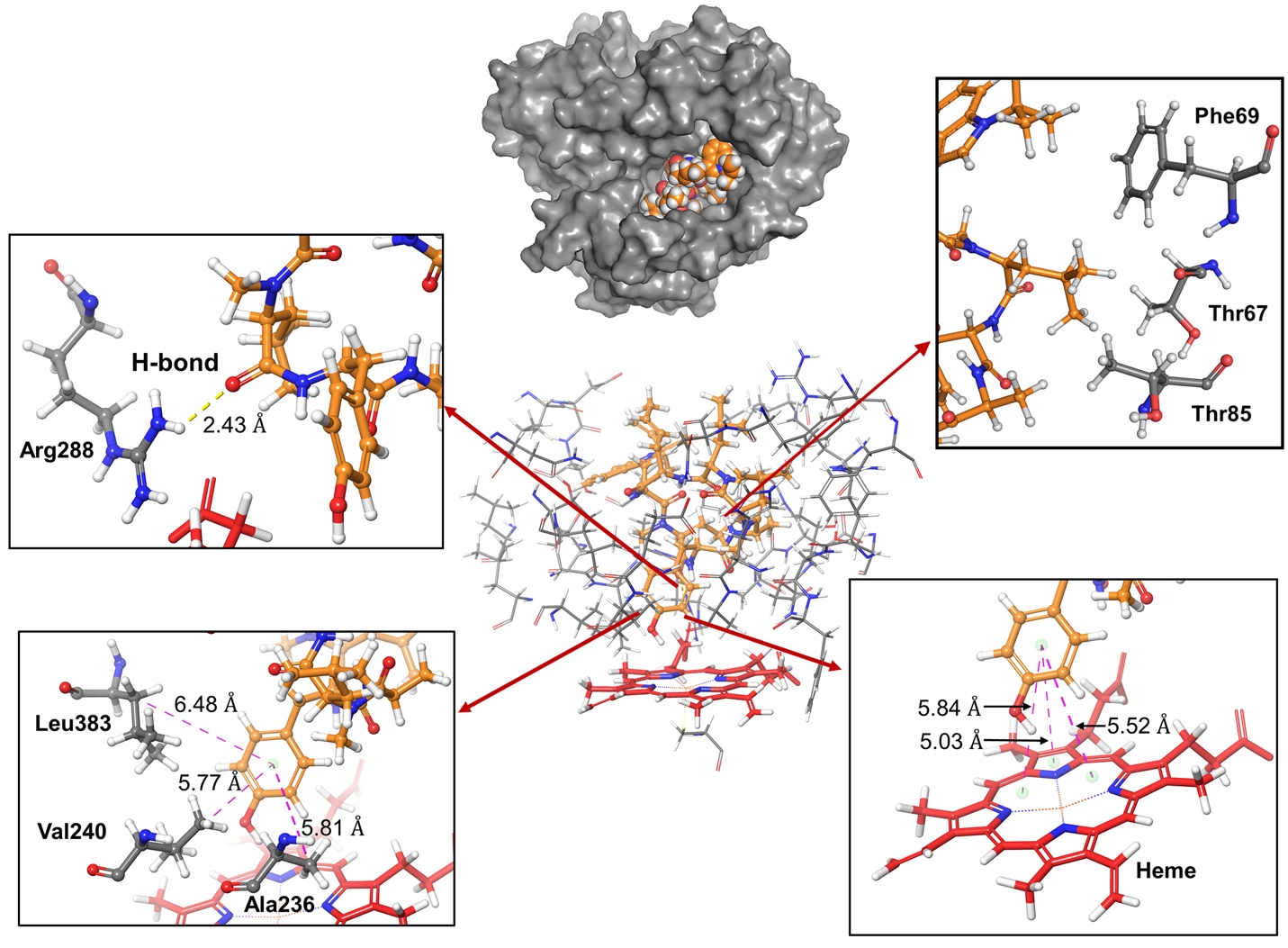


Fig. S12. Interactions between RufO and the cyclic heptapeptide shown in the docking model

The Tyr moiety of the peptide forms π–π stacking with heme and hydrophobic interactions with Ala236, Leu383, and Val240; a carbonyl group on the peptide forms the H-bond with Arg288. The Leu adjacent to the Tyr is held by Phe69, Thr67, and Thr85 through hydrophobic interactions. The heptapeptide, heme, and RufO are colored in orange, red, and gray, respectively.


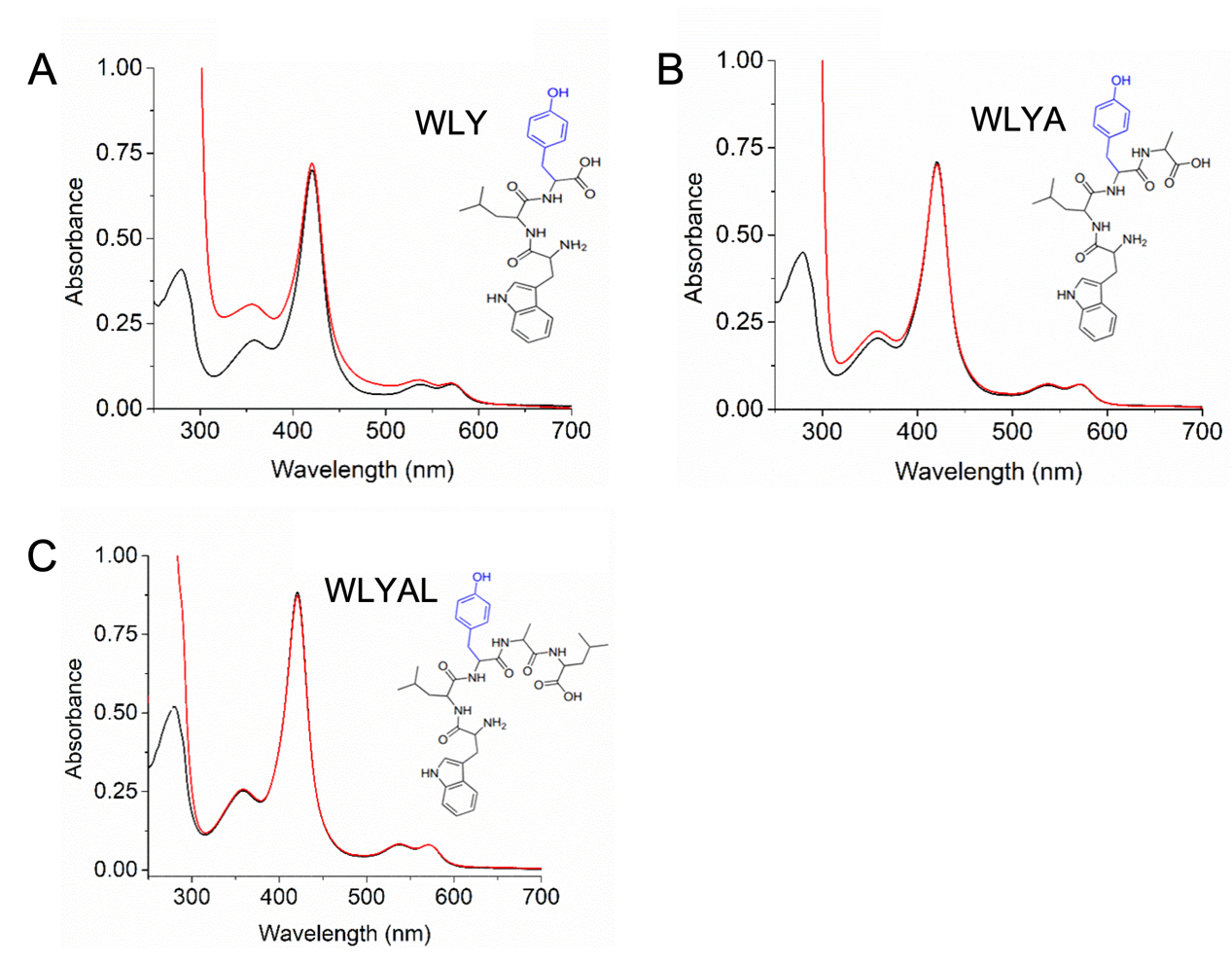


Fig. S13. UV-vis spectroscopic binding analysis of RufO with peptides WLY, WLYA, and WLYAL

UV-vis spectroscopic measurements of RufO in the absence and presence of (**A**) WLY (**B**) WLYA and (**C**) WLYAL (synthesized by GenScript). Spectra of RufO alone and RufO with the peptides are shown in black and red, respectively. For the tested peptides, no shift in the Soret and Q bands was observed, indicating a lack of binding to the active site.

**
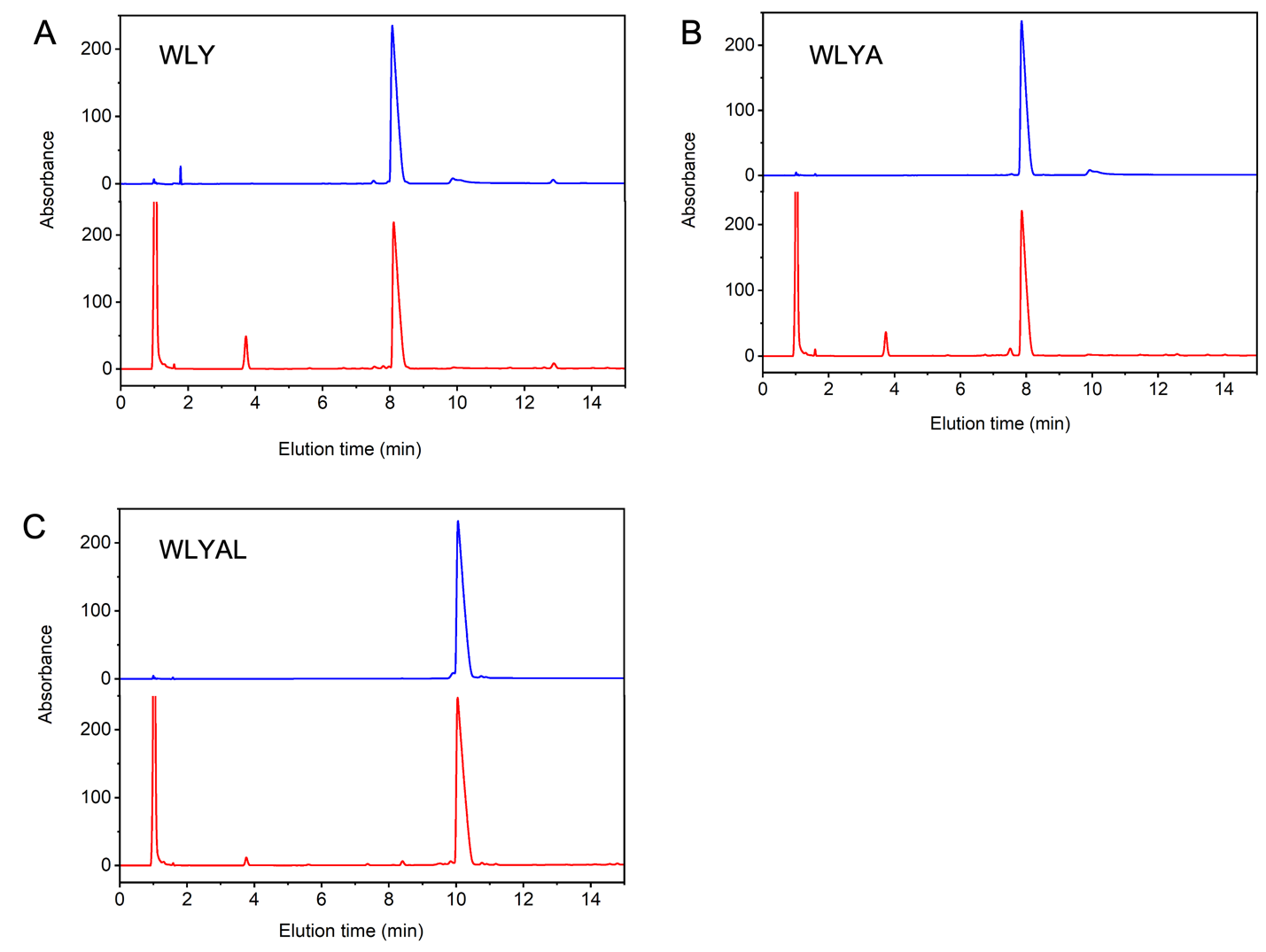
**

Fig. S14 RufO activity assays with peptides WLY, WLYA, and WLYAL

HPLC analysis of RufO activity with 1 mM (**A**) WLY, (**B**) WLYA, and (**C**) WLYAL. Peptide standards are represented in blue and enzymatic reactions are shown in red. All traces are shown with absorbance at 280 nm. No peaks corresponding to nitrated compounds (expected to elute after the starting materials) were observed in all reactions. The peaks observed with elution times before 4 min are associated with excess NADH and derivatives. The mobile phase is a gradient of 10-50% acetonitrile and 0.1% formic acid in 15 min. Other details of the HPLC method are the same as described in Experimental Procedures.

Nucleotide sequence of the codon-optimized construct of RufO

Additional 5': CAT

Additional 3': TAAAAGCTT

Cloning sites: NdeI, HindIII,

Sequence:

ATGACTGTATCACCCGCTCCAGAACACACAGACCCACTATTCTCTCCGCTGGACCCGGCAGTTCTGGCGGACCCTTATCCGGTTTATCGTCGTCTGCGCGAAACGCATCCGGTGTACTGGCACGCGGGCCTGGACAGCTGGCTTATGACCCGCCACGCTGATTGCACCGCAATTCTGCGTGATCCGGGCCGTTTCTCCACCGACTTTCGTAAGATCGATATTCCGACGCCACCGACGCTGTTGTCCCTGCAGACCTTGGACCCGCCGGACCAAACCCCGCTGCGCCACCTCGCGCTGGACGCGGTGAGAGCTCAGGATCTGGACGCCTTGCGGAAAGAGCTGACCCTGTTCGCGGACCAACTGCTCGACGAGCTGGCGGACCGTGAGTCGTTCGATTTTATCCACGATTACGCTGACGTCTTTACCTTGCGTGCAATTACCCGTTTCATCGGCGTGGAGCCGCCCGAAACTGACGAGGCCTTCGCCCGCTTCAACGATGATTTGGATCACAGCATGGATGCGCAACTGGATCCAGATGCTGAAGAGCCGGGTCTCCGTGCGCGTGCGCACTTTAACGACCTGGTGCGCAGCTGGCTGGGCGATCCTGGGCCGCATGGTGTCCTGCCGGACGTGGTTCGCCTGCTGCCGGGCAGCGGTGTTGAGGCCGATGACGTGTTGGTAAATAGCGTTCGTGCGTTTTTCCATGCGGGTTTTGAAGTTCCGTCACGTTTCCTGGGTAATGCATTGGCGGCACTTTTGGCCACCCCGGGCGCTTGGGAACAGCTAGTCCGTGGTGATGTTGGTCTGGACACCGCGGTCGAGGAGCTTATCCGCTACGTGGGTCCGGTGCAGGCACTGGCTCGCGCGTGTCTACAGGATACCGAGCTGGGCGGTATGGCAGTGAAAGAAGGTCAGGTTGTTACTGCGCTGATCGGTGCGGCTAATCGTGATCCGGATCAATTTCCGGACCCGGAAACGTTGCGTTTGGACCGCAAGCCGAACAACCACCTGGGTTTTGGTCGTGGTGCGCATAGCTGCCTGGGCCTGAACGTGGCGCGTATTGAAGCCCATGTTACCCTGGGCGCGTTGCTGCGTCACCCGGGTGTGAGAAGCGCAGGTGAACCGGTGGTTCGTCCGAACGGCACCTTGCGCGGCCTGTCTCGTCTTCCGTTGACCTTAGGC
